# Mutations in *PCYT2* disrupt etherlipid biosynthesis and cause a complex hereditary spastic paraplegia

**DOI:** 10.1093/brain/awz291

**Published:** 2019-10-22

**Authors:** Frédéric M Vaz, John H McDermott, Mariëlle Alders, Saskia B Wortmann, Stefan Kölker, Mia L Pras-Raves, Martin A T Vervaart, Henk van Lenthe, Angela C M Luyf, Hyung L Elfrink, Kay Metcalfe, Sara Cuvertino, Peter E Clayton, Rebecca Yarwood, Martin P Lowe, Simon Lovell, Richard C Rogers, Deciphering Developmental Disorders Study, Antoine H C van Kampen, Jos P N Ruiter, Ronald J A Wanders, Sacha Ferdinandusse, Michel van Weeghel, Marc Engelen, Siddharth Banka

**Affiliations:** 1 Laboratory Genetic Metabolic Diseases, Amsterdam UMC, University of Amsterdam, Department of Clinical Chemistry, Amsterdam Gastroenterology and Metabolism, Meibergdreef 9, AZ Amsterdam, The Netherlands; 2 Manchester Centre for Genomics Medicine, St Mary’s Hospital, Manchester University Hospital Foundation Trust, Health Innovation Manchester, Oxford Road, Manchester, UK; 3 Laboratory Genome Diagnostics, Amsterdam UMC, University of Amsterdam, Department of Clinical Genetics, Amsterdam Reproduction and Development, Meibergdreef 9, AZ Amsterdam, The Netherlands; 4 Institute of Human Genetics, Technical University München, Munich, Germany; 5 Institute of Human Genetics, Helmholtz Zentrum München, Neuherberg, Germany; 6 University Children’s Hospital, Paracelsus Medical University, Salzburg, Austria; 7 Division of Pediatric Neurology and Metabolic Medicine, Centre for Pediatric and Adolescent Medicine, University Hospital Heidelberg, Heidelberg, Germany; 8 Bioinformatics Laboratory, Department of Clinical Epidemiology, Biostatistics and Bioinformatics, Amsterdam Public Health research institute, Amsterdam UMC, University of Amsterdam, Amsterdam AZ, The Netherlands; 9 Division of Evolution and Genomic Sciences, School of Biological Sciences, Faculty of Biology, Medicine and Health, University of Manchester, Manchester, UK; 10 Department of Pediatric Endocrinology, Royal Manchester Children's Hospital, Manchester University Hospital Foundation Trust, Oxford Road, Manchester, UK; 11 Division of Molecular and Cellular Function, School of Biological Sciences, Faculty of Biology, Medicine and Health, University of Manchester, Manchester, UK; 12 Greenwood Genetic Center, 14 Edgewood Drive, Greenville, SC, USA; 13 Wellcome Sanger Institute, Cambridge, UK; 14 Biosystems Data Analysis, Swammerdam Institute for Life Sciences, University of Amsterdam, Science Park 904, XH Amsterdam, The Netherlands; 15 Department of (Pediatric) Neurology, Emma Children’s Hospital, Amsterdam UMC, Amsterdam, The Netherlands

**Keywords:** hereditary spastic paraplegia, PCYT2, CTP:phosphoethanolamine cytidylyltransferase, phospholipid biosynthesis, lipidomics

## Abstract

CTP:phosphoethanolamine cytidylyltransferase (ET), encoded by *PCYT2*, is the rate-limiting enzyme for phosphatidylethanolamine synthesis via the CDP-ethanolamine pathway. Phosphatidylethanolamine is one of the most abundant membrane lipids and is particularly enriched in the brain. We identified five individuals with biallelic *PCYT2* variants clinically characterized by global developmental delay with regression, spastic para- or tetraparesis, epilepsy and progressive cerebral and cerebellar atrophy. Using patient fibroblasts we demonstrated that these variants are hypomorphic, result in altered but residual ET protein levels and concomitant reduced enzyme activity without affecting mRNA levels. The significantly better survival of hypomorphic CRISPR-Cas9 generated *pcyt2* zebrafish knockout compared to a complete knockout, in conjunction with previously described data on the *Pcyt2* mouse model, indicates that complete loss of ET function may be incompatible with life in vertebrates. Lipidomic analysis revealed profound lipid abnormalities in patient fibroblasts impacting both neutral etherlipid and etherphospholipid metabolism. Plasma lipidomics studies also identified changes in etherlipids that have the potential to be used as biomarkers for ET deficiency. In conclusion, our data establish *PCYT2* as a disease gene for a new complex hereditary spastic paraplegia and confirm that etherlipid homeostasis is important for the development and function of the brain.

## Teaser

Vaz, McDermott *et al.* identify variants in *PCYT2*, which encodes a key gene in phospholipid biosynthesis, in five individuals with a new complex hereditary spastic paraplegia. Functional studies in fibroblasts and a zebrafish model confirm the pathogenic nature of the variants, while lipidomic analysis reveals potential treatment strategies and plasma biomarkers.

## Introduction

Phosphatidylethanolamine (PE) is one of the most abundant membrane lipids and is particularly enriched in the human brain where it represents ∼45% of the phospholipid fraction. In addition to its structural role in membranes, PE is involved in membrane fusion, GPI-anchor synthesis, LC3-mediated autophagy and synthesis of the brain cannabinoid receptor ligand anandamide (Vance and Tasseva, 2013). The two major biosynthetic sources of PE are the CDP-ethanolamine pathway (also known as the Kennedy pathway) and the decarboxylation of phosphatidylserine (PS) in mitochondria (Ridgway and McLeod, 2016).

**Figure 1:**
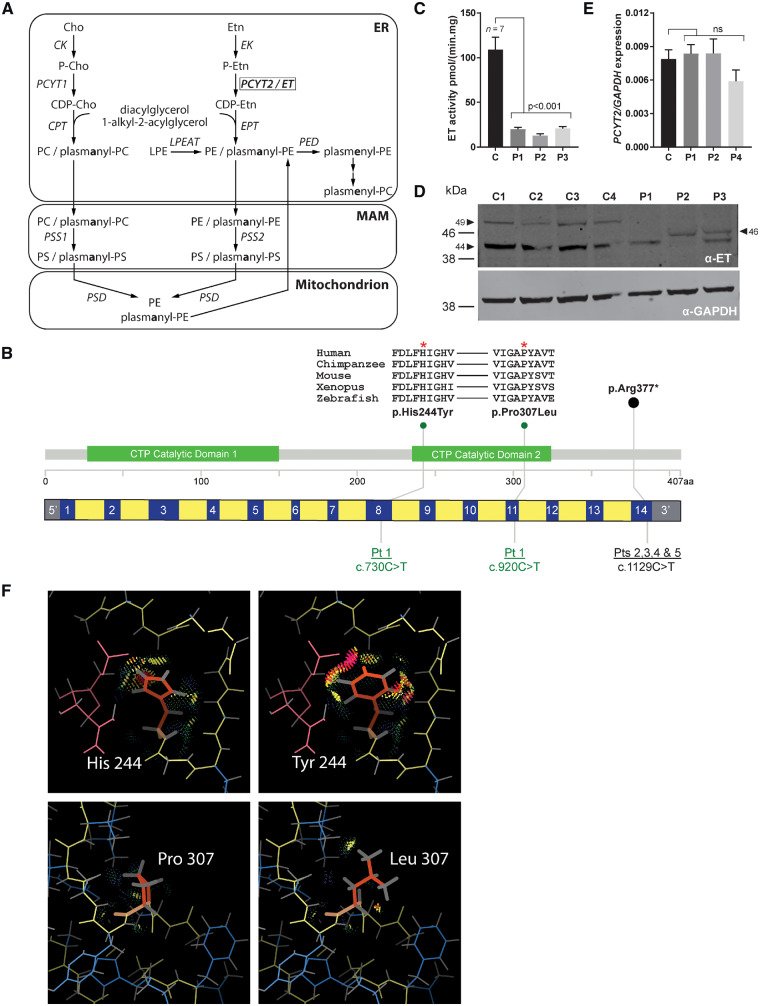
**PE-related phospholipid metabolism and functional characterization of *PCYT2* deficiency.** (**A**) Biosynthesis of phosphatidylethanolamine (PE), phosphatidylcholine (PC) and related etherphospholipids with subcellular distribution. After synthesis of phosphoethanolamine (P-Etn) by ethanolamine (Etn) kinase (EK), PCYT2/CTP:phosphoethanolamine cytidylyltransferase (ET) catalyses the conversion of CTP and P-Etn into the activated nucleotide intermediate CDP-ethanolamine (CDP-Etn). The P-Etn moiety of CDP-Etn is then transferred to the sn-3 hydroxyl of diacylglycerol by CDP-ethanolamine:1,2-diacylglycerol ethanolaminephosphotransferase (EPT) or CDP-choline:1,2-diacylglycerol choline/ethanolamine phosphotransferase (CPT) to form PE. Similarly, choline (Cho) kinase (CK) produces phosphocholine (P-Cho) that is converted to CDP-choline (CDP-Cho) by CTP:phosphocholine cytidylyltransferase (PCYT1) and condensed by CPT to form PC. PE and PC etherphospholipids are synthesized by EPT/CPT from peroxisome-derived 1-alkyl-2-acylglycerols that are condensed with CDP-Etn or CDP-choline (CDP-Cho) to form plasmanyl-PC/PE. In the mitochondria-associated membranes (MAM), base exchange of PC, PE and their corresponding plasmanyl-counterparts by PS synthase 1 and 2 (PSS1/2) yields PS and plasmanyl-PS, respectively. PS decarboxylase (PSD) that is located at the outer surface of the inner mitochondrial membrane can produce PE and plasmanyl-PE. Plasmanyl-PE is then desaturated to plasmenyl-PE (plasmalogen-PE) by plasmanylethanolamine desaturase (PED) in the endoplasmic reticulum (ER) after which plasmenyl-PC (plasmalogen-PC) is produced by base-exchange. Another source of PE is the reacylation of lyso-PE by lyso-PE acyltransferase (LPEAT). (**B**) Schematic diagram demonstrating the location of *PCYT2* variants within the gene and protein. Exons are blue, introns yellow. Patients 2–5 share a homozygous nonsense variant in the final exon. Patient 1 is compound heterozygous for two missense variants both within the second cytidylyltransferase (CTP) catalytic domain. Evolutionary conservation alignments generated using the Clustal Omega tool shows the well conserved nature of both affected amino acid residues. (**C**) ET activity in fibroblasts (mean ± SD) of controls (C) (*n* = 7) and Patients (P) 1–3 showing a strong reduction of this activity in all three patients. (**D**) ET and GAPDH western blot of fibroblast homogenate of control, Patients 1–3 (C, P1, P2 and P3) showing absence of the 49 kDa band in patients as well as reduced intensity of the normally most abundant 42 kDa band. In Patients 2 and 3, an additional band was observed at 46 kDa. (**E**) *PCYT2* mRNA expression relative to *GAPDH* (mean ± SD) for control and Patients 1, 2 and 4 (C, P1, P2, P4). *PCYT2* mRNA levels are not affected by the variants the *PCYT2* gene. (**F**) *Top*: Replacement of His244 with Tyr; *bottom*: replacement of Pro307 with Leu. Protein main chain is shown in yellow, and the mutated residue in orange, other side chains in blue and the ligand in pink. Interactions between the mutated side chain and its surrounding environment are shown with dots and spikes. Most interactions are favourable, with the exception of large van der Waals overlaps (pink); the latter are mostly between Tyr244 and the ligand.

In the CDP-ethanolamine pathway, CDP-ethanolamine is first synthesized after which it condenses with diacylglycerol (DG) to form PE. The CDP-ethanolamine pathway is also important for the synthesis of PE etherphospholipids (PE[O]) (Fig. 1A). PE[O] species are either 1-alkyl-2-acyl-PE species (plasmanyl-PE) or 1-alkyl-O-vinyl-2-acyl-PE species (plasmenyl-PEs). Plasmenyl-PEs, together with their phosphatidylcholine (PC) counterparts, are collectively called plasmalogens. Both plasmanyl- and plasmenyl-PE species are synthesized from the precursors 1-alkyl-2-acylglycerol (DG[O]). PC plasmalogens are not directly synthesized from plasmanyl-PC but are produced from PE plasmalogens where the headgroup is exchanged by choline (Fig. 1A). Plasmalogens are an important class of etherphospholipids and PE plasmalogens are by far the most abundant species (when compared to PC-plasmalogens) in the brain (Braverman and Moser, 2012). PE plasmalogens have been shown to be metabolically important for grey matter and structurally important for white matter, protecting the neuronal membrane and myelin sheath from oxidative damage (Han *et al.*, 2001). Several neurodevelopmental defects have been described where disturbed etherphospholipid metabolism is part of the pathological mechanism, including Zellweger syndrome and rhizomelic chondrodysplasia punctata (Braverman and Moser, 2012; Waterham *et al.*, 2016). Reduced levels of PE plasmalogens have also been demonstrated for Alzheimer’s disease, Parkinson’s disease, trisomy 21 and schizophrenia (Horibata *et al.*, 2018).

**Figure 2:**
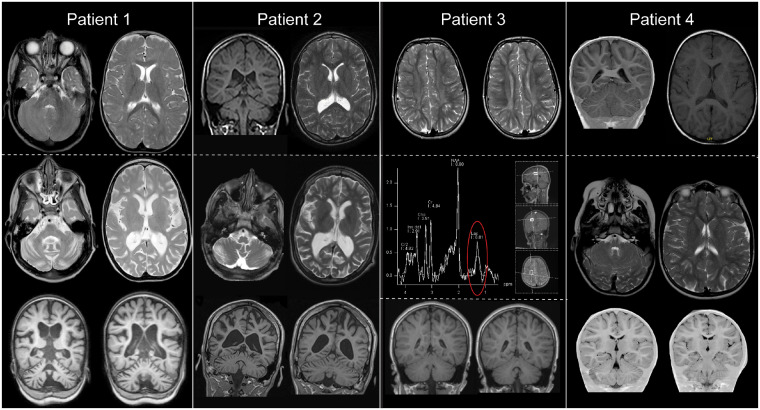
**Cranial MRI of Patients 1–4.** Patient 1: *Top* panel shows an axial T_2_-weighted MRI scan at age 8 months; scan is normal showing no structural abnormalities and myelination is appropriate for age. *Bottom* panel shows axial T_2_-weighted and coronal T_1_-weighted MRI at age 4 years and 4 months; there is prominent atrophy of supra- and infratentorial structures with enlargement of intra- and extracerebral CSF spaces. The increased signal in the cerebral white matter is aspecific and caused by axonal loss secondary to atrophy. This is mirrored in Patient 2 where the *top* panel shows a T_1_-weighted coronal section (*left*) and T_2_-weighted transverse section (*right*) at age 15 that demonstrates moderate cerebral and cerebellar atrophy, whilst the *bottom* panel shows T_2_-weighted axial sections and T_1_-weighted coronal sections at age 17, which demonstrate progression. In Patient 3, scans at age 9.5 years in the *top* panel (axial T_2_-weighted) show symmetric signal alterations of periventricular supratentorial white matter. Signal intensity of cortex and basal ganglia is normal. Compared to cranial MRI scans at age 3.5 years and 5 years (not shown) signal alterations were progressive. Magnetic resonance spectroscopy of a white matter voxel (*middle*) showed a lactate peak. The *bottom* coronal T_1_-weighted images show no structural abnormalities. In Patient 4 scans at age 3 (*top*) and age 6 (*bottom*) demonstrated no structural abnormalities.

CTP:phosphoethanolamine cytidylyltransferase (ET), encoded by the *PCYT2* gene, is the ubiquitously expressed rate-limiting enzyme for PE synthesis via the CDP-ethanolamine pathway. ET catalyses the conversion of CTP and phosphoethanolamine into the activated nucleotide intermediate CDP-ethanolamine and pyrophosphate. The phosphoethanolamine moiety of CDP-ethanolamine is then transferred to the sn-3 hydroxyl of DG or DG[O] by CDP-ethanolamine:1,2-ethanolaminephosphotransferase (EPT) to form PE or PE[O], respectively (Fig. 1A). The essentiality of ET for mammalian development is demonstrated by the fact that *Pcyt2* null mice are embryonically lethal (Fullerton *et al.*, 2007). Even the disruption of one allele of *Pcyt2* in the *Pcyt2^+/−^*mouse has major physiological effects as these animals develop insulin resistance, obesity, dyslipidaemia and liver steatosis, the hallmarks of metabolic syndrome. In these *Pcyt2^+/−^* animals, accumulation of DG leads to the production of triacylglycerols, which is fuelled by an upregulated *de novo* fatty acid synthesis (Pavlovic and Bakovic, 2013). Thus, *PCYT2* is a gene at the junction of phospholipid and neutral lipid metabolism that impacts energy homeostasis and is crucial for early development.

## Materials and methods

### Clinical and laboratory evaluation

Informed consent was obtained from the legal guardians of the subjects described in this study. All research was performed according to institutional and international guidelines for studies with human subjects and materials. See Table 1 for clinical features and Supplementary material for case reports.

**Table 1:** Clinical features of the *PCYT2* patients

	Patient 1	Patient 2	Patient 3	Patient 4	Patient 5
**General information**					
Age at assessment (years)	5.8	20	16.7	9.9	2.5
Gender	Male	Male	Male	Female	Male
Ethnicity	Hungarian	British	Turkish	US Caucasian	US Caucasian
**Mutation**					
cDNA/protein	c.920C>T & c.730C>T / p.(His244Tyr) & p.(Pro307Leu)	c.1129C>T / p.(Arg377Ter)	c.1129C>T / p. (Arg377Ter)	c.1129C>T / p.(Arg377Ter)	c.1129C>T / p.(Arg377Ter)
Consanguinity	No	Yes	Yes	Yes	Yes
**Development**					
Unassisted sitting	Not achieved	12 months	15 months	6 months	7.5 months
Independent walking	Not achieved	6 years	Not achieved	3 years	2.5 years
Speech development	Vocalization	No delay	Single words	No delay	No delay
Intellectual disability	Severe	Mild	Severe	Mild	Mild
Regression	Yes	Yes	Yes	Yes	Not reported
**Epileptic seizures**					
Age of onset (years)	2.5	16	6	5.5	2.0.
Type of seizures	TCS	TCS	FS/TCS	TCS	FS
Anti-epileptic treatment	Sodium valproate	Sodium valproate	Topiramate	Levetiracetam	None
			Levetiracetam		
			Lamotrigine, VNS		
**Examination**					
Height, cm (SDS)	n.a.^a^	142 (<<−2.5)	150 (−2.33)	121.9 (−2.47)	91.4 (−0.94)
Weight, kg (SDS)	12 (−4.5)	32 (−7.3)	50 (−1.85)	22.5 (−2.25)	13.5 (−0.44)
Head circumference (SDS)	46 (−3.3)	56 (0)	57 (0.56)	51.0 (−0.47)	49.5 (−0.36)
Dysmorphic features	No	No	No	No	No
Spasticity	Yes	Yes	Yes	Yes	Yes
Hyperreflexia	Yes	Yes	Yes	Yes	Yes
Plantar responses	Extensor	Extensor	Extensor	Extensor	Extensor
**Investigations**					
Brain MRI	Normal initially, progressive atrophy, subtle symmetric hyperintensities in cerebral white matter, MRS with voxel in affected white matter shows no lactate peak	Normal initially, progressive atrophy, subtle symmetric hypertintensities in cerebral white matter	Progressive atrophy, symmetric hyperintensities in the cerebral white matter, MRS with voxel in affected white matter shows lactate peak	Progressive atrophy, subtle symmetric hyperintensities in the cerebral white matter	Not performed
**Other features**					
Growth hormone supplementation	No	Yes	No	No	No
Ophthalmological abnormalities	n.a.	Nystagmus, poor VA	Nystagmus, optic atrophy	Nystagmus, poor VA	Nystagmus, poor VA

FS = focal seizures; MRS = magnetic resonance spectroscopy; n.a. = not available; SDS = standard deviation; TCS = tonic-clonic seizure; VA = visual acuity; VNS = vagal nerve stimulation.

a Not possible to obtain reliable measurement because of severe contractures.

### Whole exome sequencing

Trio whole exome sequencing (WES) (Patient 1 and both parents) was performed at Centogene using a Nextera Rapid Capture Exome (Illumina) for enrichment and a Nextseq or HiSeq400 platform for sequencing. Variant calling was done by their in-house pipeline. Obtained variants were prioritized using Cartagenia Bench Lab (Agilent). Variants with <5 reads, a frequency of >1% in public (ESP, dbSNP, 1KG) and/or in-house databases were excluded. *De novo*, homozygous or compound heterozygous variants present in exons or within ±6 nucleotides in the intron were evaluated, which led to the identification of two variants in *PCYT2*. Patient 2 underwent WES as part of the Deciphering Developmental Disorders (DDD) study (Deciphering Developmental Disorders Study, 2017). However, no causal variants in known developmental disorders genes were identified. Patient 2’s trio WES data were re-examined in Manchester as part of the local ‘solving the unsolved’ project (Helbig *et al.*, 2018) via a previously described pipeline and filtering strategy (Faundes *et al.*, 2018), which led to the identification of the homozygous variant in *PCYT2*. Exome sequencing for Patient 3 was performed using a Sure Select Human All Exon 50Mb bv5 Kit (Agilent) for enrichment and a HiSeq2500 as previously described (Kremer *et al.*, 2017). Reads were aligned to the UCSC human reference assembly (hg19) with BWA v.0.5.8. More than 89% of the exome was covered at least 20×. Single-nucleotide variants (SNVs) and small insertions and deletions were detected with SAMtools v.0.1.7. Variant prioritization was performed based on an autosomal recessive pattern of inheritance (MAF <0.1%). Variants absent in gnomAD and with a CADD score of 25 or more were prioritized. For Patients 4 and 5 the Agilent SureSelect^TM^ Human All Exon V5 kit was used to target the exonic regions of the genome using genomic DNA from submitted samples. The targeted regions were sequenced using the Illumina NextSeq® 500 system with 150 bp paired-end reads. Using NEXTGENe® software the DNA sequence was aligned and compared with human genome build 19 (hg19/NCBI Build 37). The filtering steps followed in both patients were similar to those described for Patient 1. The *PCYT2* variants in all patients and parents were confirmed via Sanger sequencing. Primer pairs for Sanger sequencing are available in the Supplementary material.

### Patient cell lines

We used primary skin fibroblast cell lines from anonymized healthy control subjects and the ET patients. Fibroblasts were cultured in parallel in 162-cm^2^ flasks in Ham’s F-10 medium with l-glutamine, supplemented with 10% foetal calf serum (Invitrogen), 25 mM HEPES, 100 U/ml penicillin and 100 µg/ml streptomycin and 250 µg/ml amphotericin in a humidified atmosphere of 5% CO_2_ at 37°C. After they reached confluence, the cells were harvested by trypsinization (0.5% trypsin-EDTA, Invitrogen), and washed with phosphate-buffered saline (PBS) and twice with 0.9% NaCl, followed by centrifugation at 4°C (16 100*g* for 5 min) to obtain cell pellets. Pellets were stored at −80°C until analysis.

### ET enzyme measurement in fibroblasts

Fibroblast pellets were resuspended in 0.9% NaCl and sonicated twice at 40 W. The assay had a final volume of 50 µl and consisted of (final concentrations) 10 mM TRIS buffer, pH 8.0, 10 mM MgCl_2_, 5 mM DTT, 2 mM CTP, 2 mM phosphoethanolamine and 40 µg of fibroblast protein from the homogenate. Reactions were started by addition of the homogenate and were allowed to proceed for 1 h at 37°C in a shaking water bath. Stop reagent (400 µl) (methanol + 87 pmol of ^13^C_6_-leucine) (Cambridge Isotope Laboratories) was added while vortexing to terminate the reaction. Protein precipitates were removed by centrifugation (5 min at 18 000*g*) at 4°C and the supernatants were taken to dryness under a nitrogen stream. The product of the reaction, CDP-ethanolamine, was measured by ultra-high performance liquid chromatography (UPLC) mass spectrometry (MS) as a readout of the ET activity. The residue from the enzymatic reaction was reconstituted in 100 µl of methanol/water (6:4, v/v) and 5 µl was injected into the UPLC-MS system. The chromatographic separation was performed on a Dionex Ultimate 3000 UPLC system (Thermo Fisher Scientific) with a SeQuant® Zic-cHILIC, 3 µm, 100Å, 100 × 2.1 mm PEEK coated HPLC column (Millipore) kept at 15°C. Mobile phase A consisted of 5 mM ammonium acetate in acetonitrile–water (9:1, v/v) and mobile phase B consisted of 5 mM ammonium acetate in acetonitrile–water (1:9, v/v), a linear gradient was used in 35 min to elute metabolites. The MS analysis of CDP-ethanolamine was performed on a Q Exactive™ Plus Orbitrap mass spectrometer (Thermo Fisher Scientific) with an HESI source with a spray voltage of 2.5 kV, a capillary temperature of 253°C, and the S-lens RF level set at 50.0. Data analysis was performed using Thermo Scientific Xcalibur™ software (version 4.1.50) where CDP-ethanolamine abundance was calculated using a stable isotope dilution method based on the ^13^C_6_-leucine internal standard and a CDP-ethanolamine standard curve. The amount of produced CDP-ethanolamine was used to calculate the specific activity of ET.

### Western blot

Homogenates were made from controls and patient fibroblasts using RIPA buffer (Sigma-Aldrich). The protein concentration of the homogenates was determined using the BCA Protein Assay Kit (reducing agent compatible) (Pierce). Electrophoresis was carried out using Bolt 10% Bis-Tris Plus Gels and NuPAGE® MES SDS Running Buffers (Invitrogen). For each sample, 35 μg of protein was loaded ontp the polyacrylamide gel and electrophoresed for 45 min at 120 V. The proteins were blotted onto nitrocellulose membranes using Mini iBlot® Gel Transfer Stacks Nitrocellulose (Invitrogen). After blocking non-specific binding, the membrane was incubated overnight at 4°C with specific anti-PCYT2 (ab126142, Abcam) followed by a 1-h incubation with a secondary fluorescent-labelled goat anti-rabbit antibody (IRDye 800CW LI-COR). The signal was developed using an Odyssey CLX imaging machine. The membrane was then washed with PBS-Tween 0.1% buffer. The washed membranes were incubated with anti-GAPDH (5174S, Cell Signalling) for an hour followed by incubation with a secondary fluorescent-labelled goat anti-rabbit antibody (IRDye 680CW LI-COR) and visualized as described above.

### *PCYT2* mRNA expression analysis

Total RNAs were extracted from cell pellets using RNeasy® Mini kit (Qiagen) according to the manufacturer’s protocol. RNA concentration was measured using a NanoDrop™ 2000 spectrophotometer (Thermo Scientific). RNA (1 μg) was reverse transcribed with random hexamers primer (Promega) to generate cDNA using the M-MLV Reverse Transcriptase kit (Promega), according to the manufacturer’s protocol. Quantitative real-time PCR (qRT-PCR) reactions were performed in triplicate on a Bio-Rad CFX394 Real Time system (Bio-Rad) using Power SYBR® Green PCR Master mix (Applied Biosystems).

### Zebrafish and creation of the *pcyt2* zebrafish model

Zebrafish were raised and maintained at the biological services facility at the University of Manchester under standard conditions (Westerfield, 2000). Wild-type (strain AB Notts) were bred at the University of Manchester.

Single guide (sg)RNAs were designed to target unique genomic regions within *pcyt2* exons 3 or 13 harbouring a restriction enzyme site (Supplementary material). To generate transgenic embryos, 1 μl of 20 μM EnGen NLS Cas9 protein (NEB), 500 ng/μl Cas9 mRNA, 20 ng/μl sgRNA (Sigma-Aldrich) and 0.5 μl phenol red were prepared. This mixture (1 nl) was injected into the yolk of single-cell stage zebrafish embryos using a microinjector (PLI-90 Pico-Injector, Harvard Apparatus). One hundred embryos were injected for each guide. Targeting efficiency was assessed by performing standard PCR on DNA extracted from G0 whole embryos. Regions of interest were amplified before incubation with the corresponding restriction enzyme. Samples were assessed via gel electrophoresis with the lack of a cut band demonstrating loss of the restriction site, indicating good CRISPR efficiency (Supplementary Fig. 1). Survival was measured each day for the first 5 days for the initial 100 embryos and at 6 weeks for 20 zebrafish per guide. Post-injection zebrafish were raised and maintained as described previously (Oltrabella *et al.*, 2015). Tail fin clippings were taken from surviving zebrafish at 3 months and the efficiency of the injected guides were assessed via restriction digest.

### *pcyt2* mRNA expression analysis from zebrafish embryos

For each line, 10 5-day post-fertilization zebrafish embryos were homogenized using an IKA Ultra homogenizer. Total RNA was isolated using the guanidinium thiocyanate-phenol-chloroform extraction method using TRIzol® reagent (Invitrogen). RNA (1 μg) was reverse transcribed using random hexamers to generate cDNA using SuperScript III™ First strand kit (Invitrogen) according to the manufacturer’s instructions. Oligonucleotide primers (Eurofins) for RT-PCR were designed using Primer3 (Untergasser *et al.*, 2012), primer sequences (two sets for *pcyt2*, one for loading control *eif1α*) can be found in the Supplementary material. PCR was performed on cDNA using GoTaq® green master mix (Promega).

### Extraction of phospholipids for lipidomics

Fibroblast pellets were resuspended in water and sonicated for 2 × 10 s at 8 W using a tip sonicator. Protein concentrations of the homogenates were determined using the bicinchoninic acid assay (Smith *et al.*, 1985). Phospholipids were extracted using a single-phase extraction. We added a defined amount of internal standards [0.1 nmol of CL(14:0)_4_, 0.2 nmol of BMP(14:0)_2_, 2.0 nmol of PC(14:0)_2_, 0.1 nmol of PG(14:0)_2_, 5.0 nmol of PS(14:0)_2_, 0.5 nmol of PE(14:0)_2_, 0.5 nmol of PA(14:0)_2_, 2.125 nmol of SM(d18:1/12:0), 0.02 nmol of LPG(14:0), 0.1 nmol of LPE(14:0), 0.5 nmol of LPC(14:0), 0.1 nmol of LPA(14:0), 0.5nmol of PI(8:0)_2_, 0.5 nmol DG(14:0)_2_, 0.5 nmol TG(14:0)_3_, 2.5 nmol D_3_-CE(16:0), 0.125 nmol of different sphingosines and ceramides (purchased from Avanti Polar Lipids) dissolved in 165 µl of chloroform/methanol (1:1, v/v)], and 1.5 ml of chloroform/methanol (1:1, v/v) to 1 mg protein of the fibroblast homogenates. Subsequently, the mixture was sonicated in a water bath for 10 min, followed by centrifugation at 4°C (18 600*g* for 5 min). The liquid phase was transferred to a glass vial and evaporated under a steam of nitrogen at 60°C. The residue was then dissolved in 150 µl chloroform/methanol (1:1, v/v) and 5 µl of the solution was injected for both normal phase and reverse phase HPLC-MS.

Plasma lipidomics was carried out for Patients 1–3. For Patient 3, plasma was available from two separate blood collections and both were analysed. Four replicates were extracted for each patient sample. Twenty plasma samples from (anonymous) healthy individuals were used as controls. Lipids were extracted from 20 µl plasma using the same protocol as for fibroblasts.

Each fibroblast cell line and plasma sample was analysed in quadruplicate by HPLC-MS as described below.

### Lipidomics analysis by HPLC-MS

Lipidomics analysis was essentially performed as described previously (Herzog *et al.*, 2016) using two analytical columns in two ionization modes. The first HPLC system consisted of an Ultimate 3000 binary HPLC pump, a vacuum degasser, a column temperature controller, and an auto sampler (Thermo Scientific). The lipid extract was injected onto a normal phase and a reverse phase system. The normal phase system consisted of a Luna 2 × 250 mm silica 100 Å column, 5-µm particle diameter (Phenomenex), the column temperature was maintained at 25°C. Phospholipids were separated from interfering compounds by a linear gradient between solution B (chloroform/methanol, 97:3 v/v) and solution A (methanol/water, 85:15, v/v). Solution A contained 0.125 ml formic acid and 0.25 ml of 25% (v/v) aqueous ammonia per litre of eluent, solution B contained 0.125 ml formic acid per litre. The gradient (0.3 ml/min) was as follows: 0–1 min, 10% A; 1–4 min, 10% A–20% A; 4–12 min, 20% A–85% A; 12–12.1 min, 85% A–100% A; 12.1–14.0 min, 100% A; 14–14.1 min, 100% A–10% A; and 14.1–15 min, equilibration with 10% A. All gradient steps were linear, and the total analysis time, including the equilibration, was 15 min. A Q Exactive™ Plus (Thermo Scientific) mass spectrometer was used in the negative and positive electrospray ionization mode. In both ionization modes, mass spectra of the lipid species were obtained by continuous scanning from m/z 150 to m/z 2000 with a resolution of 280.000. Nitrogen was used as the nebulizing gas. The spray voltage used was 2500 V (−) and 3500 V (+), and the capillary temperature was 256°C. S-lens RF level: 50, auxiliary gas: 10, auxiliary gas temperature: 300°C, sheath gas: 50, sweep cone gas: 2. The reverse phase system consisted of an Acquity UPLC HSS T3 100 × 2 mm column, 1.8 µm particle diameter (Waters). The column temperature was maintained at 60°C. A linear gradient between solution B (2-propanol/methanol, 90:10 v/v) and solution A (methanol/water, 40:60, v/v) was used. Both solutions contained 0.1% formic acid (v/v) and 10 mM ammonium formate. The gradient (0.4 ml/min) was as follows: 0–1 min, 100% A–80% A; 1–16 min, 80% A–0% A; 16–20 min, 0% A; 20–20.1 min, 0% A–100% A; and 20.1–21 min, equilibration with 100% A. All gradient steps were linear, and the total analysis time, including the equilibration, was 21 min. A Q Exactive™ Plus mass spectrometer was used in the negative and positive electrospray ionization mode. In both ionization modes, spectra were obtained by continuous scanning from m/z 150 to m/z 2000 with a resolution of 280.000. Nitrogen was used as the nebulizing gas. The spray voltage used was 3700 V (−) and 3100 V (+), and the capillary temperature was 360°C. S-lens RF level: 50, auxiliary gas: 12.5, auxiliary gas temperature 350°C, sheath gas: 50, sweep cone gas: 2.

### Bioinformatics and statistical analysis of lipidomics data

The raw LC/MS data were converted to mzXML format using MSConvert (Chambers *et al.*, 2012). The dataset was processed using an in-house developed metabolomics pipeline written in the R programming language (http://www.r-project.org). In brief, it consisted of the following five steps: (i) preprocessing using the R package XCMS (Smith *et al.*, 2006) with minor changes to some functions to better suit the Q Exactive™ data; notably, the definition of noise level in centWave was adjusted and the stepsize in fillPeaks; (ii) identification of metabolites using an in-house database of (phospho)lipids, with known internal standards indicating the position of most of the lipid clusters, matching m/z values within 3 ppm deviation; (iii) isotope correction to obtain deconvoluted intensities for overlapping peak groups; (iv) normalization on the intensity of the internal standard for lipid classes for which an internal standard was available and scaling on measured protein content per sample; and (v) statistical analysis, visualization and interpretation of the data. The statistical programming language R was used to analyse the lipidomics data. Different statistical comparisons were made including Student’s *t*-test, one-way ANOVA with *post hoc* Bonferroni correction to search for relevant changes between patients and controls. Lipids were ranked for differential abundance based on their variable importance of projection scores. The variable importance of projection scores was constructed using partial least squares regression discriminant analysis using the R package mixOmics (Thévenot *et al.*, 2015). Heat maps of metabolites were created using the R programming language package gplots. Colour in the heat maps reflects the logarithm of the relative metabolite abundance with red being higher and blue lower than the mean abundance value per metabolite. Summation of relative abundances of same class lipids to calculate total phospholipid levels was carried out with the assumptions of equal response to their respective internal standard and are by no means comparable between different species to compare relative concentrations. Only comparisons within the same species can be made between different sample groups (e.g. control versus patient). Data in figures are presented as mean ± standard deviation (SD).

### Statistics

**Figure 3:**
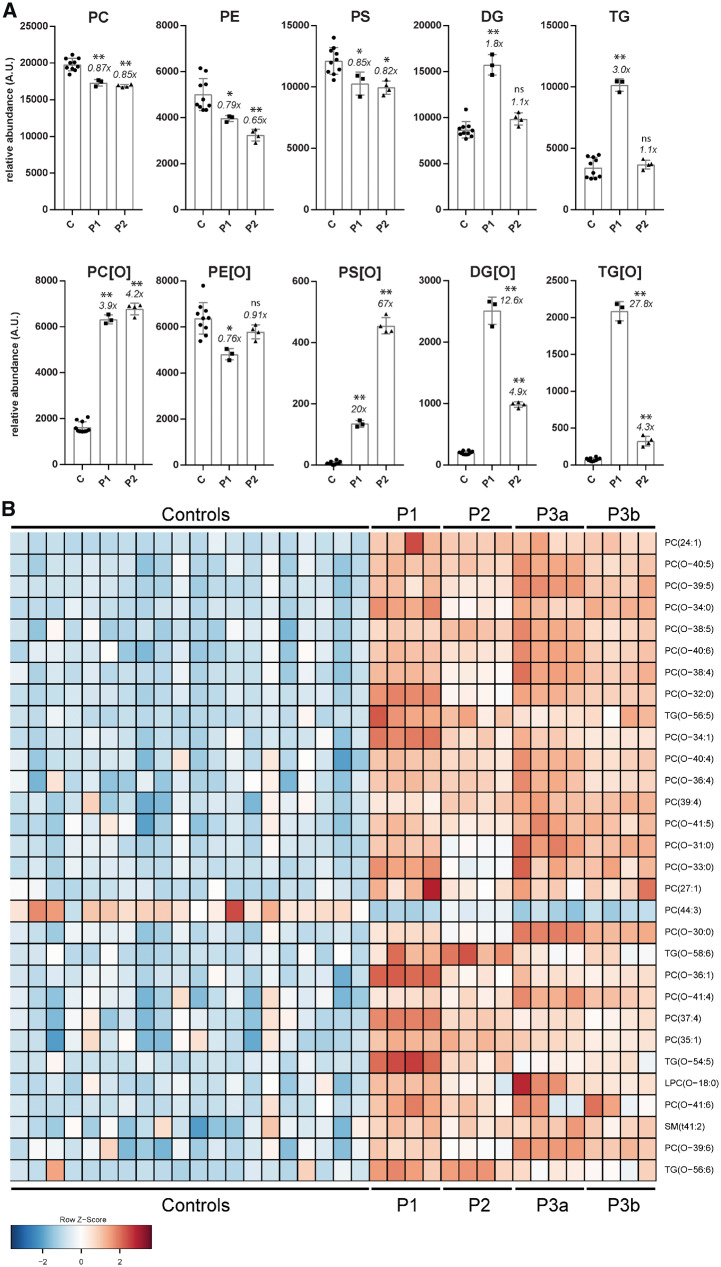
**Lipidomics in *PCYT2* deficiency.** (**A**) Summation of lipidomic species per major class for controls (three controls each measured as *n* = 3), Patients 1 (*n* = 3) and 2 (*n* = 4), mean ± SD is shown and *x*-fold difference of the patient mean compared to that of control subjects. *P < 0.01, ^**^P ≪ 0.001. (**B**) Lipidomics in plasma: heat map of the top 30 lipids ranked according to VIP score (measure of a variable’s importance in the PLS-DA model). Plasma was available from Patients 1–3 (the latter from two separate blood collections, designated 3a and 3b). The lipidome of the three patients shows a clear accumulation of PC[O] and TG[O] species. ns = not significant; TG = triacylglycerol; TG[O] = 1-alkyl-2,3-diacylglycerols.

**Figure 4:**
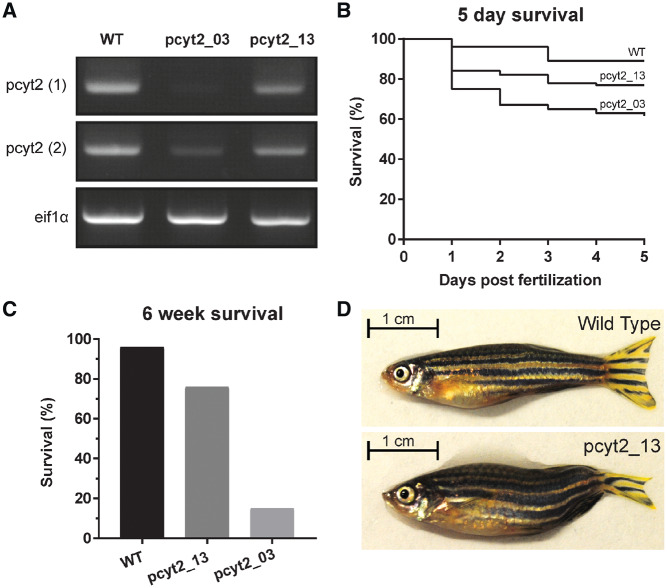
**CRISPR zebrafish *PCYT2* model.** Two transgenic zebrafish lines were created by knocking out the third (pcyt2_03) and final 13th exon (pcyt2_13), respectively. (**A**) Complementary DNA expression analysis of *pcyt2* using two primer sets in wild-type (WT), pcyt2_03 and pcyt2_13 showing low/absent *pcyt2* expression in pcyt2_03 and moderately reduced *pcyt2* expression in pcyt2_13. *eif1α* was used as loading control. (**B**) Kaplan-Meier plot for the survival of the first 5 days post-fertilization, significance was calculated using the log rank and Wilcoxon test [χ^2^ for equivalence of death rates: wild-type versus pcyt2_03 = 21.440258 (*P* < 0.0001), wild-type versus pcyt2_13 = 6.497151 (*P* = 0.0108) and pcyt2_03 versus pcyt2_13 = 4.700852 (*P* = 0.0301)]. (**C**) Survival at 6 weeks post-fertilization for wild-type, exon 3 and exon 13 deletion showing a significantly higher survival for the exon 13 deletion mutant, significance calculated via fisher exact test (*P* < 0.001). (**D**) The pcyt2_13 line compared with the wild-type at 6 weeks of age showing smaller overall size and abnormal tail-fin morphology.

Graphad Prism 7.03 was used to perform statistical comparisons. For Figs 1C, E and 4A where controls and patients were compared, an ANOVA was carried out followed by a Dunnett’s multiple comparisons test. For the Kaplan-Meier plot in Fig. 3, comparing the survival of the first 5 days post-fertilization, significance was calculated using the log rank and Wilcoxon test (χ^2^ for equivalence of death rates).

### Data availability

The authors confirm that the data supporting the findings of this study are available within the article and/or its Supplementary material. Upon reasonable request, raw genetic data can be made available. 

## Results

### Genetic analysis

Through international collaboration we identified five individuals, from four families, with rare and predicted protein damaging biallelic *PCYT2* (NM_001184917.2) variants (GeneMatcher) (Sobreira *et al.*, 2015) (Fig. 1B) (see Table 1 and Supplementary material for detailed case reports, family pedigrees and WES variant lists).

Patient 1 was compound heterozygous for two missense variants, c.730C>T (p.His244Tyr) and c.920C>T (p.Pro307Leu). Both variants are predicted to result in substitution of highly conserved residues affecting the C-terminal cytidylyltransferase domain of *PCYT2* (Fig. 1B) and are predicted to be damaging by multiple *in silico* tools (Supplementary material). The p.Pro307Leu variant is absent from population databases whereas the p.His244Tyr variant has a minor allele frequency of 0.000016 (http://gnomad.broadinstitute.org/variant/17-79864636-G-A; accessed on 16 January 2019), and has never been observed in homozygous state. The p.His244Tyr variant affects the nucleotide-binding motif HxGH and has previously been shown to prevent formation of Pcyt2α and -β hetero- and homodimers, critical for ET activity, in mice (Tie and Bakovic, 2007; Pavlovic *et al.*, 2014*a*).

Four individuals from three unrelated families (Patients 2–5) shared the homozygous nonsense variant c.1129C>T (p.Arg377Ter) (Fig. 1B and Table 1). This variant is located in the last exon of 8 of 12 known human *PCYT2* transcripts, including the canonical NM_001184917.2 transcript (Supplementary Fig. 3) and is predicted to cause truncation of both catalytically active ET-α and ET-β isoforms. This variant has a minor allele frequency of 0.0001182 in individuals of non-Finnish European descent (http://gnomad.broadinstitute.org/variant/17-79862804-G-A; accessed on 16 January 2019) but has never been observed in the homozygous state. Furthermore, no loss-of-function *PCYT2* variants have ever been observed in the general population in a homozygous state. Sanger sequencing traces of Patients 1–3 are available in Supplementary Fig. 4. Other variants found in Patients 1–3 are available in the Supplementary material. Of note the siblings, Patients 4 and 5, shared a rare homozygous missense *SACS* c.11249A>G p.(Asn3750Ser) variant of uncertain significance. Pathogenic *SACS* variants cause autosomal recessive spastic ataxia of Charlevoix-Saguenay (ARSACS; OMIM 270550). However, these patients did not have the cranial MRI features or the characteristic retinal striations seen in ARSACS. Furthermore, the *SACS* variants were predicted to be tolerated according to SIFT and PolyPhen-2 (data not shown). 

### Clinical features of *PCYT2* patients

All five individuals presented with global developmental delay in the first year of life (Table 1 and Supplementary material). Developmental impairment ranged from mild (ambulatory and verbal communication) to severe (unable to sit unsupported and no verbal communication). During follow-up all patients showed a progressive course of disease with loss of previously acquired skills. There were no consistent dysmorphic features or organomegaly. All patients developed a progressive spastic para- or tetraparesis and epilepsy with focal and/or tonic-clonic seizures with onset between 2 and 16 years of age. Nystagmus and poor visual acuity were reported in Patients 2–5, Patient 3 had optic atrophy. Where available, serial brain MRIs showed progressive cerebral and cerebellar atrophy (Fig. 2), Patient 1 had corresponding microcephaly.

### Functional consequences of the *PCYT2* variants at the mRNA, enzyme and structural level

We explored the effect of the *PCYT2* variants on ET activity in fibroblasts of controls and Patients 1 (with compound heterozygous missense variants), 2 and 4 (both with the nonsense variant). This showed that the ET activity was significantly decreased, but not absent, in all patient fibroblasts (Fig. 1C). To identify the reason for the decrease in enzyme activity, we performed western blots with ET antibody in fibroblast lysates obtained from controls and Patients 1–3. ET-specific bands in controls were observed at apparent molecular masses of 42 and 49 kDa. The 49 kDa band was absent in all three patients, but in Patients 2 and 3 a band at an apparent molecular mass of ∼46 kDa was seen (Fig. 1D and Supplementary Fig. 5 for the complete western blot). The intensity of the 42 kDa band was reduced in Patients 1 and 3 and almost absent in Patient 2. Next, we performed RT-PCR on mRNA extracted from puromycin-treated and untreated cultured fibroblasts of these patients. This showed no significant reduction in *PCYT2* transcript levels (Fig. 1E) suggesting that the variants do not affect mRNA expression. Next, we assessed the effect of the missense variants on protein structure. This showed that His244 is in the active site and makes contact with the substrate (Fig. 1F). The p.His244Tyr replacement results in the mutated side chain making van der Waals overlaps with the substrate, probably disrupting binding and/or catalysis. The predicted ΔΔGmut is +3.6 kcal/mol, additionally suggesting that the protein structure may be less stable. Pro307 was found to be in a surface loop and the p.Pro307Leu replacement removes the close complementarity of the proline with the rest of the protein (Fig. 1F). The predicted ΔΔGmut for this change is +1.8 kcal/mol, again suggesting a less stable protein structure. It was not possible to model the nonsense variant as the known solved ET enzyme structure does not include the C-terminus of the protein.

### Lipidomics in fibroblasts and plasma

Because of the role of ET in phospholipid biosynthesis, we performed lipidomic analyses in fibroblasts of Patients 1 and 2. In patient lipidomes we detected a significant accumulation of PC etherphospholipid species (PC[O] and LPC[O]), neutral lipid species [DG and triacylglycerols (TG)] and neutral etherlipid species including 1-alkyl-2,3-diacylglycerols (TG[O], the etherlipid equivalents of triglycerides) and 1-alkyl-2-acylglycerols (DG[O], the etherlipid equivalents of diacylglycerols). In addition, we found reduced levels of PC species [PC and lysophosphatidylcholine (LPC)], PE, lysophosphatidylethanolamine (LPE) and their etherphospholipid analogues (PE[O] and LPE[O]) (Fig. 3A, Supplementary Fig. 6 and Supplementary material). The ET deficiency thus had profound effects on the lipidome, especially on etherphospholipid metabolism. We, therefore, investigated the nature of the accumulating TG[O] species by tandem mass spectrometry to ascertain whether these were plasmanyl or plasmenyl species in order to better understand their origin. This showed that the TG[O] species were almost exclusively alkyl-diacyl (equivalent to plasmanyl) and not alk-1-enyl-diacyl (equivalent to plasmenyl), as observed previously (May *et al.*, 1992). This indicates that these species are directly synthesized from accumulating DG[O] as a result of the ET deficiency.

To understand the effects of the ET deficiency on plasmalogen synthesis we also investigated whether the accumulating PE[O] and PC[O] species were plasmalogens (plasmenyl species) or non-plasmalogen etherphospholipids (plasmanyl species). This was done by comparing the levels of representative PE[O] and PC[O] species with/without hydrochloric acid treatment. This treatment hydrolyses the plasmenyl but not the plasmanyl species on the sn-1 position. Comparison of treated and untreated samples showed that PC[O] species consisted entirely of plasmanyl-PC and no plasmenyl-PCs (PC-plasmalogens) could be detected in both patients and controls. In contrast, and quite surprisingly, all PE[O] species consisted of plasmenyl-PEs (PE plasmalogens), again both in patients and controls (Supplementary Fig. 7). The relatively normal PE plasmalogen levels in the absence of ET activity prompted the search for evidence of an alternative way for their production. In a previously reported route via the PS decarboxylase pathway, where PC[O] species are converted via plasmanyl-PS[O] into PE[O] (Xu *et al.*, 1991, 1994) (Fig. 1A), PS[O] species are obligatory intermediates. Indeed, PS[O] species could be detected in patients, where Patient 2 showed higher levels than Patient 1, but were low/absent in control subjects, indicating that the PS-decarboxylase route is used to synthesize plasmalogens in ET deficiency. The identity of the PS[O] species as plasmanyl-PS was confirmed by MS/MS analysis of two PS[O] species (Supplementary Fig. 8 and Supplementary material).

In addition to the effects on etherphospholipids we found that in ET-deficient fibroblast there was a trend towards deficiency of polyunsaturated fatty acid (PUFA)-containing species in PE, PS and phosphatidylinositol (PI). Levels of species with 38 or more combined carbon atoms in the fatty acid side chains and with five or more double bonds [e.g. PS(40:5), which could represent a PS with C18:0/C22:5 or C20:1/C20:4] were less abundant in ET deficiency (Supplementary Fig. 9 and Supplementary material).

Finally, to investigate potential biomarkers for this disease in an easily accessible body fluid we performed plasma lipidomics in 20 control subjects and three *PCYT2* patients (Fig. 3B). As in fibroblasts, we detected significant accumulation of PC[O] etherphospholipids in plasma of patients accompanied with a variable degree of TG[O] accumulation (Supplementary material).

### Zebrafish pcyt2 model

*Pcyt2* ^−/−^ mice are embryonically lethal and no animal models with hypomorphic variants have been described. A single orthologue of human *PCYT2* is present in zebrafish. We used the CRISPR-Cas9 system to create two distinct *pcyt2* knockout zebrafish lines, one targeting exon 3 (pcyt2_03) and the other targeting the final exon 13 (pcyt2_13) (Supplementary Fig. 1 and Supplementary material). Messenger RNA expression analysis of injected zebrafish embryos at 5-days post-fertilization demonstrated reduced expression in the pcyt2_03 injected models when compared with wild-type and pcyt2_13 embryos (Fig. 3A). The pcyt2_03 fish displayed significantly lower survival of G0 zebrafish in comparison with pcyt2_13 at 5 days (Fig. 4B). Long-term survival assessed at 6 weeks revealed an even more striking pattern with 17% and 80% survival in G0 pcyt2_03 zebrafish and pcyt2_13, respectively, when compared to a 96% survival rate in uninjected wild-type zebrafish (Fig. 4C). Genotyping via restriction digest carried out on the DNA extracted from the tail-fins of three surviving pcyt2_03 fish showed that the restriction site was not lost in one fish, indicating that the CRISPR-Cas9 system had not induced a mutation. In the other two there was evidence of mosaicism. One of these fish was significantly smaller than the other injected and uninjected fish. Similarly, the genotyping of the six surviving pcyt2_13 fish showed that the CRISPR-Cas9 system had generated mutations in all but one fish (Supplementary Fig. 2). The surviving mutated pcyt2_13 zebrafish were smaller than their uninjected counterparts and had consistent abnormal tail-fin morphology (Fig. 4D).

## Discussion

We describe five individuals with a complex hereditary spastic paraplegia (cHSP) and biallelic *PCYT2* variants. The pathogenicity of these variants is supported by multiple lines of argument including absence of the variants in homozygous state in population databases, high evolutionary conservation of the substituted residues (Fig. 1B) and *in silico* modelling of variants indicating disruption of binding or catalysis or loss of protein stability (Fig. 1F). This was further supported by our observation of significant reduction of ET enzyme activity (Fig. 1C) and reduced ET protein levels (Fig. 1D) in patient-derived fibroblasts, and profound changes in the lipidomic profiles of patient fibroblasts and blood (Fig. 3).

Of the three variants detected in this study, two were missense and one was a nonsense variant in the last exon which, as proven via mRNA analysis (Fig. 1E), escapes nonsense-mediated decay and therefore could still produce protein products. We observed noticeable differences in the patterns of protein expression between the controls and patients (Fig. 1D). However, it is apparent that there are detectable levels of ET protein in the patient cells, which provide a possible explanation for the ET rest activity that was found in the enzyme activity assay. The explanation of the differences in the protein expression pattern remains unclear but as *PCYT2* produces at least three isoforms of differing molecular weights as a result of alternative splicing (Tie and Bakovic, 2007; Pavlovic *et al.*, 2014*b*) these could represent different ET isoforms. Particularly, all patient cells demonstrated absent or severely depleted protein at the level of 49 kDa band, which could represent the longer isoform, PCYT2-α. In both patients with the p.Arg377Ter mutation (Patients 2 and 3), a band at 46 kDa level was observed that may represent truncated PCYT2-α isoform (Fig. 1D). The levels of the most abundant 44 kDa band were also clearly reduced when compared to control subjects, especially in Patient 2, which could represent the shorter PCYT2-β isoform.

At the mRNA level, similar findings were observed in G0 zebrafish models generated via CRISPR-Cas9 system induced knockout of exon 13, the final zebrafish *pcyt2* exon, resulting in preserved mRNA expression when compared to knockout of exon 3 and wild-type controls. We detected significantly lower survival rates at both 5 days and 6 weeks of age in the exon 3 knockout G0 zebrafish when compared to the exon 13 knockout. Of the few exon 3 knockout animals that survived, genotyping demonstrated either incomplete or absent knockout via the CRISPR-Cas9 system, indicating that complete deletion of *pcyt2* in zebrafish may not be compatible with life. The surviving zebrafish from the group of animals in which the final exon 13 was targeted were smaller than their uninjected counterparts and had consistent abnormal tail-fin morphology (Fig. 4D). Our zebrafish results should be considered as preliminary and only supportive evidence to the human findings described here. This is because the experiments are subject to efficiency of the CRISPR-Cas9 system, which frequently produces variable degrees of mosaicism in G0 fish. Stable germline mutants will be required for a thorough investigation in the future. Interestingly, similar morphological defects have been observed in another zebrafish model of HSP caused by loss-of-function variants in *GBA2* supporting the validity of our observations (Martin *et al.*, 2013). Notably, *Pcyt2*^−/−^ complete knockout mice are embryonically lethal (Fullerton *et al.*, 2007). We therefore conclude that the disease-causing human *PCYT2* variants found here are likely to be hypomorphic and that in vertebrates, complete loss of ET function may be incompatible with life.

Lipidomics showed that ET heavily impacts etherlipid metabolism and indicated that in ET deficiency, PE and etherphospholipids are mainly produced via the alternative PS decarboxylase pathway. However, this compensatory pathway may be insufficient in the case of complete loss of ET activity, or ET might have an additional role in metabolism that cannot be compensated by the PS decarboxylase pathway.

Although the individuals were identified based on their genotypes, reverse phenotyping (de Goede *et al.*, 2016) demonstrated remarkable phenotypic convergence adding evidence that the deleterious *PCYT2* variants are causal for the patients’ phenotype. All patients presented with global developmental delay and pyramidal tract signs, which were more marked in the lower extremities. There was clear progression over time, with increasing spasticity and a regression in cognitive abilities. Progressive cerebral and cerebellar atrophy was also observed in patients where MRI scans were available. A diffuse increase in signal was noted on T_2_-weighted images on the MRI of all patients, which likely reflects progressive axonal loss rather than a primary white matter disorder. In leukodystrophies, the signal changes are usually more pronounced and follow specific patterns. Here, the changes are mostly subtle and diffuse (with deep periventricular predominance), accompanied by clear atrophy (especially in Patients 1 and 2). This pattern (profound atrophy and subtle increases of signal on T_2_-weighted images) is more compatible with a neuronal disorder (Schiffmann and van der Knaap, 2009) than a leukodystrophy. The findings on magnetic resonance spectroscopy are inconclusive. While Patient 3 showed a clear lactate peak in a white matter voxel (Fig. 2E), this was not reproduced in Patient 1 (data not shown). The significance of this finding is unclear at this time. All patients presented with epilepsy, nystagmus and poor visual acuity. Four of five patients additionally showed growth delay. Based on clinical features (with clear ‘cortical’ features like epilepsy and intellectual disability) and MRI findings, ET deficiency is characterized by progressive neuronal loss and essentially appears to be a ‘grey matter disorder’ and can be classified as a complex hereditary spastic paraplegia. ET is expressed across most human tissues, although there is some variation in the distribution of the PCYT2α and PCYT2β isoforms, with the canonical PCYT2α transcript typically predominant (www.proteinatlas.org accessed on 2 June 2019). There is no preferential expression of ET in the CNS comparative to other tissues, but the highest concentration of etherphospholipids is found in the brain (Brites *et al.*, 2004). Therefore, the effects of reduced ET activity may be particularly obvious in the CNS, suggesting why our patients presented with a predominantly neurological phenotype.

Recently, *SELENOI* variants were reported in two families with a progressive neurodegenerative disorder characterized by mild intellectual disability, spasticity, epilepsy, progressive atrophy with increased signal in the periventricular white matter, a phenotype strikingly similar to the patients described in this paper (Ahmed *et al.*, 2017; Horibata *et al.*, 2018). *SELENOI* encodes EPT1, which catalyses the final step in the CDP-ethanolamine pathway (Fig. 1A), the step directly after the ET reaction.

Our results, together with those found for EPT1 deficiency, suggest that the CDP-ethanolamine pathway is crucial for the development and function of the CNS and that primary or secondary changes in phospholipid metabolism can lead to a progressive grey matter disorder. In line with these findings, several other progressive neurodegenerative spastic paraplegia disorders have been associated with variants in genes involved in lipid metabolism, including *FA2H*, *GBA2* and *DDHD2* (Edvardson *et al.*, 2008; Schuurs-Hoeijmakers *et al.*, 2012; Martin *et al.*, 2013). Biochemically, we found metabolic changes that were consistent with the location of the metabolic block in the phospholipid biosynthesis pathway. ET deficiency causes a shortage of CDP-ethanolamine leading to accumulation of DG and DG[O], which in turn are shunted towards triacylglycerols and TGs[O]. The changes in the lipidome are similar to those found in the murine *Pcyt2* model, but differ in some aspects. *Pcyt2^+/^*^−^ mice developed features of metabolic syndrome including liver steatosis because accumulating DG is shunted towards triacylglycerol synthesis (Singh *et al.*, 2012). In the liver of *Pcyt2^+/^*^−^ animals there was a general deficiency of polyunsaturated fatty acids (PUFAs) due to the upregulated triacylglycerol synthesis and the concomitant synthesis of saturated fatty acids (Fullerton *et al.*, 2007). In fibroblasts of ET patients, a deficiency of PUFAs was also seen to a certain degree for PE, PS and PI (Supplementary Fig. 8). Our patients did not develop features of metabolic syndrome but this remains to be confirmed in future studies. Despite biochemical similarities with respect to accumulating metabolites and mechanisms, the phenotype of the ET patients is different from the homozygous (embryonic lethal) and heterozygous *Pcyt2* mouse models. This may be due to the differences in residual activity of ET, which is 0% in the homozygous knockout mice, 15–20% in the fibroblasts of ET patients and 65–80% in the heterozygous mice due to upregulation of the expression of the *Pcyt2* remaining allele.

In addition to the changes found in neutral lipid metabolism, synthesis of etherphospholipids was disturbed as shown by the massive accumulation of PC[O]/LPC[O] species as the accumulating DG[O] condenses with CDP-choline via the unaffected CDP-choline route. Surprisingly, the PE[O]/LPE[O] levels were somewhat decreased, but not deficient, in contrast to what would be expected from the classic phospholipid synthesis pathways. This indicates that plasmanyl-PC is converted to plasmanyl-PS by PS synthase 1 (PSS1), after which plasmanyl-PS is decarboxylated to form plasmanyl-PE, which is then converted to plasmenyl-PE (Fig. 1A). Isotope labelling experiments in mouse heart, kidney and liver previously showed that this pathway is not active in these tissues (Arthur and Page, 1991); however, in glioma cells similar labelling experiments showed that PE/PC-plasmalogens can be synthesized via PS (Xu *et al.*, 1991, 1994). In addition, the ET deficiency likely generates considerable metabolic pressure to synthesize plasmenyl-PE via plasmanyl-PS because of the accumulating precursors (DG[O]→PC[O]→PS[O]→PE[O]). Also, cultured cells have been shown to be highly dependent on the PS decarboxylation pathway for PE synthesis (Vance and Tasseva, 2013), which would further channel the accumulating metabolites into this alternative synthetic pathway for PE plasmalogens. This suggests that the PS decarboxylase rescue route is highly important to allow PE etherphospholipid and plasmalogen biosynthesis, especially in case of ET deficiency. Interestingly, the levels of PS[O] in fibroblasts were higher in Patient 2 when compared to Patient 1 whereas all other metabolic changes were more severe in Patient 1. This might reflect a less efficient use of the PS decarboxylase pathway as a rescue option, possibly explaining the more severe phenotype in Patient 1. In summary, our lipidomics analyses reveal that ether lipid metabolism, including neutral etherlipids and etherphospholipids, is critically dependent on ET activity.

Similarly, PC[O] accumulation, PE[O]/PE plasmalogen levels reduction and lower levels of PUFA-containing PS species have also been shown in patients with EPT1 deficiency, the enzyme directly downstream of ET. In fibroblasts of both ET and EPT1 deficiency, DG[O] accumulates because of the blocked CDP-ethanolamine pathway. Consequently, DG[O] is shunted into the CDP-choline pathway thereby resulting in increased PC[O] levels. In both disorders, there is compensation via other synthetic pathways to synthesize PE[O]/PE plasmalogens (likely via the PS decarboxylase route) but PE etherphospholipids are lower and there are changes in polyunsaturated species of different major classes (Ahmed *et al.*, 2017; Horibata *et al.*, 2018). This underscores the common lipid disturbance in both defects. Plasmenyl-PE species are crucial in the structural maintenance of neuronal membranes and the myelin sheath. ET-deficient fibroblasts demonstrated a moderate reduction in PE and PE[O] species compared to control samples, with levels presumably partially corrected via the mitochondrial PS decarboxylase pathway. Ideally, investigation of brain material of patients should be done to confirm the impact of the ET deficiency on phospholipid metabolism. Still, the reduction in PE and PE[O] species may be sufficient to disrupt the proper maintenance and development of the CNS, leading to the progressive phenotype observed in the ET patients. Alternatively, the large accumulation of intermediate species (DG, DG[O], triacylglycerols, TG[O], PC[O]) seen in the ET patients may be independently damaging. In a mouse model for spastic paraplegia type 54 (OMIM 615033), biallelic *DDHD2* variants led to a significant accumulation of triacylglycerols in the CNS. This triacylglycerol accumulation correlated with an increase in amounts and size of lipid droplets within neurons when compared to wild-type mice, where lipid droplets are rarely seen (Inloes *et al.*, 2018). Accumulation of lipid droplets within the CNS is potentially neurotoxic and has been seen previously in neurodegenerative conditions such as Alzheimer’s disease (Gómez-Ramos and Asunción Morán, 2007; Liu *et al.*, 2017), suggesting that the neutral lipid accumulation in ET deficiency could play a causative role in the degenerative phenotype.

The phospholipid imbalance in ET deficiency suggests a higher demand for choline, the metabolite crucial to allow the synthesis of plasmanyl/plasmenyl species, but also serine, which is required for the synthesis of PE, plasmanyl and plasmenyl PE[O] and PC plasmalogen species via the PS decarboxylase rescue route. Supplementation of these metabolites could possibly support the rescue routes and allow some restoration of the lipidomic imbalance. Interestingly, supplementation with serine stabilizes patients with disorders of serine biosynthesis. These disorders are characterized by profound global developmental delay, intractable seizures and severe, progressive microcephaly and abnormal phospholipid composition (Ferreira *et al.*, 2018; Glinton *et al.*, 2018). The possibility of treatment makes early diagnosis of ET deficiency crucial, which necessitates the identification of biomarkers in easily accessible fluids. Our lipidomic studies of patients’ plasma indicates that PC[O] and TG[O] accumulation are potential biomarkers for ET deficiency and possibly also for other disorders of the CDP-ethanolamine pathway such as EPT1 deficiency. These biomarkers can also be potentially used to clarify variants of uncertain significance in genes in this pathway, which further emphasizes the complementary roles of genomics and biochemistry in accurate diagnosis and management of patients (Ghosh *et al.*, 2017).

In summary, using genomics, lipidomics, *in vitro* and *in vivo* studies we describe a novel autosomal recessive inborn error of biosynthesis of complex lipids caused by hypomorphic *PCYT2* variants resulting in a complex hereditary spastic paraplegia. Our findings highlight the importance of the CDP-ethanolamine pathway, and specifically of ET, for the synthesis of ether(phospho)lipids and brain lipid metabolism.

## Supplementary Material

awz291_Supplementary_Data

## Abbreviations

DG =: diacylglycerol
DG[O] =: 1-alkyl-2-acylglycerol
ET =: CTP:phosphoethanolamine cytidylyltransferase
(L)PC =: (lyso)phosphatidylcholine
(L)PC[O] =: (lyso)phosphatidylcholine etherphospholipid
(L)PE =: (lyso)phosphatidylethanolamine
(L)PE[O] =: (lyso)phosphatidylethanolamine etherphospholipid
PS =: phosphatidylserine
PS[O] =: phosphatidylserine etherphospholipid
