# Supplementary material for: Mutations in PCYT2 disrupt etherlipid biosynthesis and cause a complex hereditary spastic paraplegia
Source: Brain. 2019 Oct 22;142(11):3382–97. doi: 10.1093/brain/awz291 (PMC6821184; doi:10.1093/brain/awz291)
Supplement: awz291_Supplementary_Data [file awz291_supplementary_data.zip › awz291-Suppl_data/Supplementary_Data2.pdf]

SUPPLEMENTARY INFORMATION 1: Case reports and DNA analysis information

Contents:

1. **Clinical reports**
2. **Pedigrees of the 4 families**
3. **Primer pair table for confirmatory Sanger sequencing of variants**
4. **Results of variant analysis by different in silico prediction tools**
5. **WES variantlist**

1. Clinical reports

Patient 1

Patient 1, a 5½ year old boy, is of Hungarian descent. He was born at term after an unremarkable pregnancy. At the age of about 7 months a developmental delay became apparent, as patient was unable to sit unsupported. At that time he was able to roll over, reached for toys and was communicative (made eye contact and smiled at his parents). In the following years there was a clear regression, he lost the ability to roll over and no longer reached for objects. There is little spontaneous movement. His parents noted “jerky” eye movements. At the age of 2.5 years he developed tonic-clonic seizures. After starting valproic acid the seizures are reasonably well controlled. Currently, he is completely ADL dependent. He is not tube fed, but food has to be mashed. There is no verbal communication. On examination there were no dysmorphic features and no abnormalities on general examination. There is a clear microcephaly. Patient is alert, makes sounds, but no recognizable words. There is no gaze palsy, no nystagmus during examination. There is profound spasticity of upper and lower limbs, with very little spontaneous movement. The thumbs are adducted and flexed, the hands are closed. There are contractures in shoulder, elbows, hips, knees and ankles. The tendon reflexes were brisk and bilateral Babinski signs (triple response) were present. Extensive diagnostic testing was done over the years. Initial MRI of the brain was normal, but follow-up scans at the age of 2 and 4 years show progressive cerebral and cerebellar atrophy. A CGH array was normal. Extensive metabolic testing in plasma and urine revealed no abnormalities. Whole exome sequencing (trio analysis) initially revealed no causative variants. However, re-analysis led to the identification of compound heterozygous mutations in *PCYT2* (c.920C>T & c.730C>T / p.(His244Tyr) & p.(Pro307Leu)).

Patient 2

Patient 2, a 20 year old man, is an offspring of first cousin consanguineous parents of White British decent. He was born at term after an unremarkable pregnancy weighing 3.13kg (9th-25th centile) with an occipitofrontal circumference (OFC) of 37cm (91st-98th centile) and a length of 49cm (9th-25th centile). His neonatal period was unremarkable.

He achieved the ability to sit unsupported at 11 months. At 15 months he developed an intention tremor which significantly impaired fine motor skills. At 18 months he developed bilateral lateral gaze nystagmus. Ophthalmological assessment did not show any retinal abnormalities. He achieved independent walking at 3 years. At 7 years of age he had developed truncal ataxia, poor balance, marked nystagmus and staccato speech. Although still independently mobile, he required a walking aid. His ambulation was increasingly limited by bilateral spasticity, most marked in the legs. His mobility gradually deteriorated further throughout his teenage years. He was given a trial of baclofen, but no response in leg spasticity was noted to this therapy. By age 19 years he became wheelchair dependent. On the most recent examination at age 20 years he had increased tone in all four limbs, particularly marked in the legs with severe contractures. Power was normal in the upper limbs (MRC 5/5) but there were only flickers of movement in the lower legs (MRC 1/5). Reflexes were globally brisk in all four limbs with marked clonus and upgoing plantar reflexes bilaterally. He had past pointing, demonstrated dysdiadochokinesia, cerebellar or "staccato" speech and truncal ataxia requiring adaptations to his wheelchair. He had lateral gaze pendular nystagmus bilaterally and his visual acuity had deteriorated significantly, requiring size 70 font to read.

From an intellectual perspective, he coped well in a mainstream primary school, achieving within the average range. However, gradually he started falling behind his peers at school and towards the later teenage years worsening short term memory and a mild cognitive decline were noted. At age 16 years he had two generalised tonic-clonic seizures and he was commenced on sodium valproate by a consultant neurologist. He has not had any subsequent seizures.

An MR brain scan at 2 years of age was entirely normal. By age 18 years however, as his clinical condition deteriorated, an MR brain demonstrated generalised atrophy with loss of white matter and marked thinning of the corpus callosum (Figure 2).

Postnatal growth restriction was noted during infancy with his height and weight falling below the 0.4th centile by 12 months. His weight and height has remained below the 0.4th centile whilst his head circumference fell below the 2nd centile. Endocrine investigations at 8 years of age revealed low peak growth hormone with low IGF-1 (IGF1: 40µg/L (Normal range for age 55-240) and a low Growth hormone peak during a stimulation test (12.5mU/L (Normal >15mU/L)). A repeat brain MR at this stage

demonstrated a small anterior pituitary. He was commenced on growth hormone and initially responded well to a dose of 0.58mg per day, growing by 6 cm in the first year. He maintained a stable growth velocity and spontaneously entered puberty at 13 years of age. His height has remained below the 0.4th centile throughout treatment. At age 19 years his height and OFC were both below the 0.4th centiles; he remains on growth hormone supplementation. Radiographs of his lower limbs showed a mild form of non-rhizomelic chondrodysplasia punctate.

Karyotype analysis and 8x60K array (Oxford Gene Technology, UK) comparative genomic hybridization were both normal. Long chain fatty acids, phytanic acid, red cell plasmalogens and dihydroxyacetone phosphate acyltransferase testing were all unremarkable at 3 years of age. Screening for the mucopolysaccharidoses, Fredrich's ataxia and the spinocerebellar ataxia syndromes type 1,2,3,6,7 and 8 revealed no abnormalities. The family subsequently underwent trio Whole Exome Sequencing (WES) as part of the Deciphering Developmental Disease Study (DDD) (1) that did not identify any causal variants in any known developmental disorder genes. The trio exome data was re-examined as part of a local 'solving the unsolved' project (2) using a previously described pipeline (3), which led to identification of a biparental homozygous nonsense *PCYT2* (NM_0011849917 C.1129C>T p.Arg377*) variant. An additional rare *de novo* missense variant *NUTM1* (NM_175741:c.559G>A p.Gly187Arg) was also detected in this individual. Two additional rare biallelic *ARMC10* (NM_001161009.2:c.423+1G>C) and *CLIP4* (NM_024692.5:c.1399+35_1399+36insT) variants were detected. No rare X-Linked variants were detected.

Patient 3

Patient 3, a 17-year-old adolescent, is the first offspring of first cousin consanguineous parents of Turkish decent. He was born at term after an unremarkable pregnancy and delivery (APGAR 9/10/10) weighing 3.65 kg (69th centile) with an occipitofrontal circumference (OFC) of 36 cm (73th centile) and a length of 50 cm (21st centile). His postnatal adaptation and neonatal period were unremarkable. While his development was considered as normal during the first months of life, bilateral nystagmus and delayed motor development were observed both at age 7 months. Despite physicotherapy according to Vojta starting at age 8 months, he did not catch up, but still made some progress in the beginning. He achieved the ability to sit unsupported at 15 months, learn to crawl with 24 months and to stand with aid with 36 months, while independent walking was never achieved. Fine motor skills were less significantly impaired. Except for a few single words, he did not learn to speak. Furthermore, he developed severe intellectual disability, aggravating with age.

During infancy general muscular hypotonia with normal power in upper and normal limbs and normal tendon reflexes was prominent, while muscular tone increased with age and he developed progressive bilateral spastic diparesis with hyperreflexia. His ambulation, mostly by crawling and rolling, was increasingly limited and he also lost other motor skills in the following years. He developed bilateral sensorineural hearing loss with 4 years, and optic nerve atrophy at age 6 years. Therapy-resistant epilepsy started at age 6 years with focal seizures that tended to secondarily generalize. Despite start of antiepileptic therapy (at last, levetiracetam, topiramate, and lamotrigine) he never became seizure-free, however, seizure control was transiently improved by implantation of a vagus nerve stimulator at age 14 years.

On the most recent examination at age 17 years he is wheel-chair bound and otherwise immobile, and requires support for all his daily activities. Spastic diparesis has further deteriorated, now also involving the upper limb and including dystonic elements. He has developed hip flexion contracture and scoliosis as well as short stature (height 150 cm [2nd percentile], weight 50 kg [3rd percentile], head circumference 57 cm [3rd percentile]). His movements, particularly with his hands, are stereotypic. He does not establish contact with other people and vocalizes randomly.

Cranial MRI studies (at last, age 9.5 years) showed symmetric signal alterations (T2w and FLAIR) of white matter, specifically at periventricular supratentorial white matter, around the 4th ventricle, in tegmentum pontis, upper cerebellar peduncles and bilateral medial nuclei dentate. MR spectroscopy unravelled a lactate peak in the white matter. The described changes were slightly progressive compared to MRI studies performed at age 3.5 and 5 years.

Whole exome sequencing revealed rare, non-synonymous variants in 48 genes. From this list variants in four genes were prioritized based on the absence or rarity in GnomAD and a CADD score of 25 or higher (*UBAP1L*, *ACAN*, *FTSJ3*, *PCYT2*). This included the *PCYT2* homozygous c.1129C>T (p.Arg377Ter) (NM_001184917.2) variant.

Patients 4 and 5

Patient 4 is a 9½-year-old female who was product of a term vaginal delivery from an 18-year-old primigravida mother and 22-year-old father. The parents are second cousins once removed. Routine obstetric ultrasounds during the pregnancy were normal. She was delivered in vertex presentation with a birth weight of 3.55 kg, a length of 52 cm, and a head circumference of 33 cm. Her parents noted that she had abnormal movements of her eyes when she was a toddler. Ophthalmology evaluation confirmed that she had nystagmus and severe vision impairment. She was diagnosed with a seizure disorder at age

5½ years and began treatment with levetiracetam. She was noted by her neurologist to have spastic diplegia and was prescribed baclofen. She was diagnosed with global developmental delay and a mixed receptive and expressive language disorder at age 6 years.

Her developmental history reveals that she rolled at 5 months, sat at 6 months and crawled at 8 months however, she did not walk until 3 years of age. Truncal hypotonia and peripheral hypertonia were present and have progressed. She has received physical and occupational therapy for several years. She has indistinct, dysarthric speech and requires speech therapy.

Physical examination at age 6 years revealed a height of 105.5 cm 3rd percentile and head circumference of 50.3 cm 10th to 25th percentile. She had mild flattening of the nasal bridge and mild facial hypotonia. The upper extremities demonstrated normal tone the lower extremities had significant hypertonia and hyperreflexia with bilateral ankle clonus.

She was seen for follow-up at age 7½ years and was noted to have stable vision but a rotary nystagmus. Her seizures were well-controlled. She had decreased coordination of her hands but still had purposeful movement. She continued to have spastic diplegia. She had a 2-3 year delay in her cognitive abilities and continued to receive physical occupational and speech therapies. Growth parameters continued to track at the 3rd percentile for height and the 10th percentile for head circumference. She continued to have nystagmus, truncal hypotonia, and peripheral hypertonia with clonus.

She was stable when seen for follow-up at age 8½ years with nystagmus and peripheral hypertonia. She had good use of her upper extremities and was able to feed herself. She continued to have cognitive delays but was making progress in her development. Her head circumference was 50.9 cm (25th percentile). She demonstrated a scissoring gait when walking with assistance.

At age 9.9 years her exam showed mild progression of her lower extremity hypertonicity, hyperreflexia, and spasticity. Her upper extremities appeared to mildly involved and her dysarthria had progressed.

She had a normal karyotype. Microarray analysis revealed several large areas with loss of heterozygosity but no deletions or duplications were detected. She then had whole exome sequencing which lead to identification of a homozygous nonsense *PCYT2* (NM_0011849917 C.1129C>T p.Arg377*) variant. Another rare homozygous missense *SACS* c.11249 A>G p.(Asn3750Ser) variant of uncertain significance was also identified. Pathogenic *SACS* variants cause autosomal recessive spastic ataxia of Charlevoix-Saguenay (ARSACS; OMIM#- 270550) (4). However, the proband did not have the characteristic cranial MRI features including pontine abnormalities and cerebral atrophy and also did not have the characteristic retinal striations seen in ARSACS.

At age 7 months her brother (patient 5) presented with milder but similar symptoms including hypertonia of his legs and nystagmus. Molecular testing revealed that he was also homozygous for the *PCYT2* and *SACS* gene variants seen in his sister. Examination at 18 months showed normal growth parameters and a fine rotary nystagmus, and hypertonia, hyperreflexia, and spasticity of the lower extremities with ankle clonus. Follow-up examination at age 2.9 years showed typical growth with length, weight, and head circumference of 91 cm, 13.5 kg, and 49.5 cm, respectively. He continued to have nystagmus, and hypertonia, spasticity, hyperreflexia, clonus of the lower extremities. The upper extremities were currently unaffected.

Bibliography

(1). Deciphering Developmental Disorders Study. Prevalence and architecture of de novo mutations in developmental disorders. *Nature* 542, 433–438 (2017).

(2). Helbig, K. L. *et al.* De Novo Pathogenic Variants in CACNA1E Cause Developmental and Epileptic Encephalopathy with Contractures, Macrocephaly, and Dyskinesias. *The American Journal of Human Genetics* 103, 666–678 (2018).

(3). Faundes, V. *et al.* Histone Lysine Methylases and Demethylases in the Landscape of Human Developmental Disorders. *The American Journal of Human Genetics* 102, 175–187 (2018).

(4). Synofzik et al. Autosomal recessive spastic ataxia of Charlevoix Saguenay (ARSACS): expanding the genetic, clinical and imaging spectrum. Orphanet Journal of Rare Diseases 2013, 8:41-54 (2013).

2. Pedigrees of the 4 families

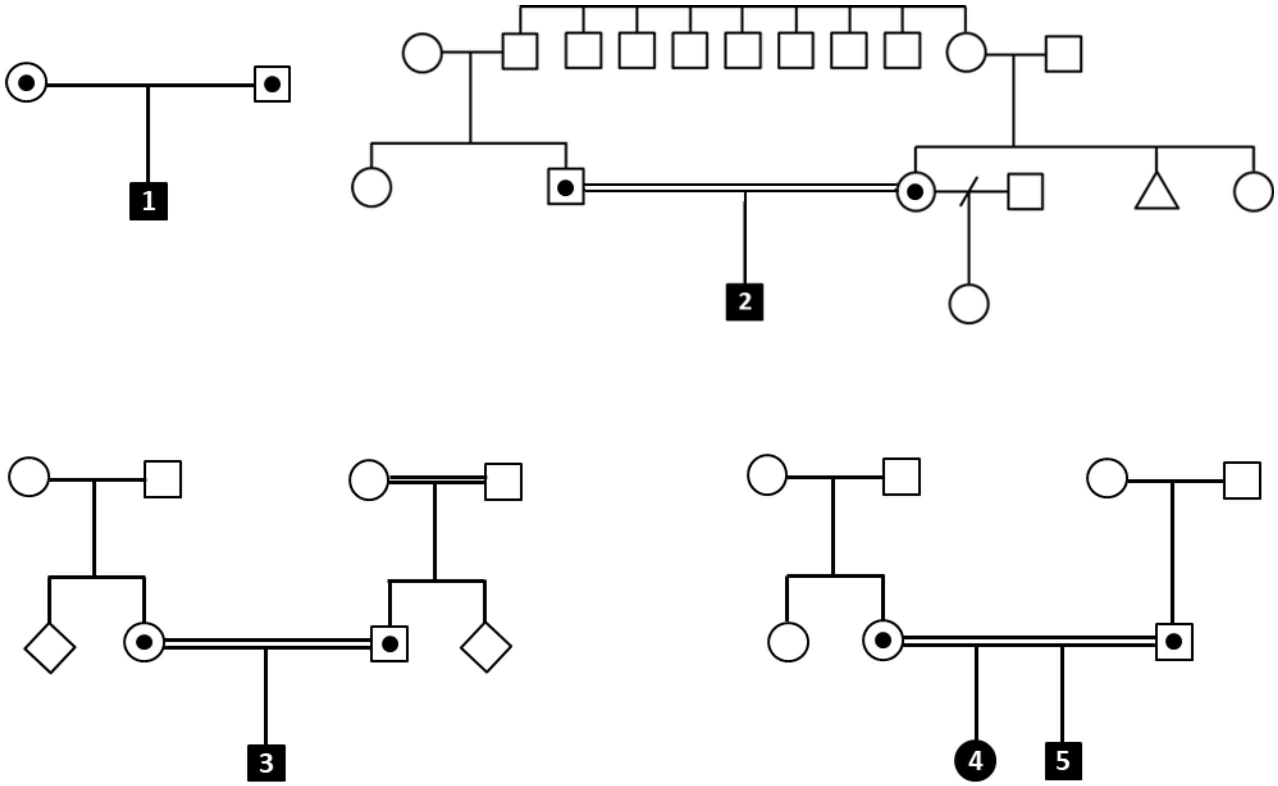

3. Primer pair table for confirmatory Sanger sequencing of variants

cPatient	Variant	Forward Primer	Reverse Primer
1	PCYT2 c.730C>T	GTGACAGCTGAGGGTTTGGT	CTGACAGCTCATGCCCAAG
	PCYT2 c.920C>T	CCATCCCTGCTACTCACTGG	CACAGCCAGCCTGCATTC
2	PCYT2 c.1129C>T	AGCAACCTCACCACAGACCT	CAAGGAGGCAGAGTCCTCAC
3	PCYT2 c.1129C>T	AGGGCATCAGATGGGTCTTG	TCTCTGAGCAGCTTTGCTGG
4	PCYT2 c.1129C>T	AGCAACCTCACCACAGACCT	CAAGGAGGCAGAGTCCTCAC
5	PCYT2 c.1129C>T	AGCAACCTCACCACAGACCT	CAAGGAGGCAGAGTCCTCAC

4. Results of variant analysis by different *in silico* prediction tools

Patient	Transcript	Variant	Effect on Protein	Grantham Score	Mutation Taster	PolyPhen2	SIFT
1	NM_001184917.2	c.730C>T	p.(Pro307Leu)	93	Disease Causing	0.997/1.0	0
		c.920C>T	p.(His244Tyr)	98	Disease Causing	1.0/1.0	0
2	NM_001184917.2	c.1129C>T	p.(Arg377Ter)	N/A (Truncating)	Disease Causing	N/A	N/A
3	NM_001184917.2	c.1129C>T	p.(Arg377Ter)	N/A (Truncating)	Disease Causing	N/A	N/A
4	NM_001184917.2	c.1129C>T	p.(Arg377Ter)	N/A (Truncating)	Disease Causing	N/A	N/A
5	NM_001184917.2	c.1129C>T	p.(Arg377Ter)	N/A (Truncating)	Disease Causing	N/A	N/A

Table demonstrating the PCYT2 variants identified in the study and the *in-silico* tools that were used to predict their pathogenicity. The homozygous variants in patients 2-5 were automatically called as pathogenic by these models because of their truncating effect. Both missense variants in patient 1 were predicted to be damaging by all of the *in silico* tools used.

5. WES variantlist

Patient	Transcript	Gene	c.DNA	Protein	zygosity
**1**	NM_004564.2	GATB	c.1198-6del		homozygous
	NM_004473.3	FOXE1	c.532_537del	p.A178_A179del	homozygous
	NM_001018088.2	VPS13C	c.3234T>C	p.=	homozygous
	NM_006141.2	DYNC1LI2	c.1379-4del		homozygous
	NM_001271604.2	JPH3	c.467_472dup	p.A156_A157dup	homozygous
	NM_014927.3	CNKSR2	c.651A>G	p.=	homozygous
	NM_001136234.1	SUPT20HL1	c.1534_1551del	p.A512_A517del	homozygous
	NM_001013736.2	FAM47C	c.1869_1904dup	p.E624_P635dup	homozygous
	NM_152869.3	RGN	c.136A>G	p.T46A	homozygous
	NM_001178099.1	ZNF182	c.795C>T	p.=	homozygous
	NM_000555.3	DCX	c.1099G>T	p.A367S	homozygous
	NM_178813.5	AKAP14	c.386C>G	p.A129G	homozygous
	NM_152692.4	C1GALT1C1	c.666G>C	p.Q222H	homozygous
	NM_016267.3	VGLL1	c.298G>A	p.A100T	homozygous
	NM_017514.4	PLXNA3	c.1958C>T	p.P653L	homozygous
	NM_017514.4	PLXNA3	c.4077C>T	p.=	homozygous
	NM_004369.3	COL6A3	c.1778A>G	p.E593G	heterozygous
	NM_004369.3	COL6A3	c.1562C>T	p.S521L	heterozygous
	NM_001256879.1	POLD2	c.354C>T	p.=	heterozygous
	NM_001256879.1	POLD2	c.171C>T	p.=	heterozygous
	NM_000603.4	NOS3	c.2460G>A	p.=	heterozygous
	NM_000603.4	NOS3	c.2479G>A	p.V827M	heterozygous
	NM_001282203.1	PCYT2	c.632C>T	p.P211L	heterozygous
	NM_001282203.1	PCYT2	c.442C>T	p.H148Y	heterozygous
	NM_130444.2	COL18A1	c.549C>T	p.=	heterozygous
	NM_130444.2	COL18A1	c.3673C>T	p.R1225W	heterozygous
**2**	NM_0011849917	PCYT2	c.1129C>T	p.Arg377*	Homozygous
	NM_001161009	ARMC10	c.423+1G>C	N/A	Homozygous
	NM_024692.5	CLIP4	c.1399+35_1399+36insT	N/A	Homozygous
	NM_175741	NUTM1	c.559G>A	p.Gly187Arg	Hetrozygous
	NM_001304	CPD	c.3613C>T	p.Leu1205Phe	Homozygous
	NM_020877	DNAH2	c.7411G>A	p.Val2471Met	Homozygous
	NM_020223	FAM20C	c.951_952insGACAGGTGAGCCCTTCC TTCCTCCCTCCATCCGC	p.Ile320fs	Homozygous
	NM_017439	GSAP	c.1121-7delT	N/A	Homozygous
	NM_001124758	SPNS2	c.1607+6_1607+7insGG	N/A	Homozygous
	NM_139276	STAT3	c.2228G>T	p.Gly743Val	Homozygous
**3**	NM_001163692.1	UBAP1L	c.710C>G	p.Pro237Arg	Homozygous
	NM_002861.3	PCYT2	c.1075C>T	p.Arg359Ter	Homozygous
	NM_004970.2	IGFALS	c.1708G>A	p.Asp570Asn	Homozygous
	NM_017647.3	FTSJ3	c.2327G>A	p.Arg776Gln	Heterozygous
	NM_017647.3	FTSJ3	NM_017647.3:c.1345G>C	p.Asp449His	Heterozygous
**4/5**			Not available		
			Not available		
