# Supplementary material for: Mutations in PCYT2 disrupt etherlipid biosynthesis and cause a complex hereditary spastic paraplegia
Source: Brain. 2019 Oct 22;142(11):3382–97. doi: 10.1093/brain/awz291 (PMC6821184; doi:10.1093/brain/awz291)
Supplement: awz291_Supplementary_Data [file awz291_supplementary_data.zip › awz291-Suppl_data/Supplementary_Data3.pdf]

Supplemental information 2 - CRISPR zebrafish guide information

Pcyt2 03 - Exon 3 Guide Design

CAGTTACGCCAGGCCAAGGCGATGGGAGATTATCTGGTCGTAGGAGTTCACACAGACGgtaagcttcacacttcatgtttcttcact
ataatgctgtaataatatgtaatgaggcattgtttttcaccttcag[AGGAAATAGCCAAGCACAAGGGTCCTCCAGTCTTCACACA
GGCAGAGCGTTATAAAATGATACGGGCCATCAA[x]GTGGGTGGATGAGATCGTGGAAGGGGCTCCGTATGTTACTACGCTGGAGACGC
TGGACAAGTACAACTGCGATTTCTGCGTGCACGGAG]gtgaggatccgttacacgctgcatatcagtagccgtctgtaactctgtag
ctcagtcggcctagggtttgaactgcaaggtcgttggtttgaattccaggaaaggcaagtactgataataatgtgcctcttgagtgc
aaggcaagtctctgctgccaaatgcactgcactaatgttaacgtcatatccttaaccctttatagggcactcgttggagtgttactg
gggttatgagaagact

Primer Pair #1 (Pink)

OLIGO	start	len	tm	gc%	any_th	3'_th	hairpin	seq
LEFT PRIMER	1	19	51.52	42.11	0.00	0.00	0.00	gtaagcttcacacttcatg
RIGHT PRIMER	286	20	55.05	45.00	0.00	0.00	0.00	tacagagttacagacggcta

SEQUENCE SIZE: 477
INCLUDED REGION SIZE: 477

PRODUCT SIZE: 286, PAIR ANY_TH COMPL: 0.00, PAIR 3'_TH COMPL: 0.00

Primer Pair #2 (Yellow)

OLIGO	start	len	tm	gc%	any_th	3'_th	hairpin	seq
LEFT PRIMER	31	20	54.71	45.00	0.00	0.00	0.00	TATCTGGTCGTAGGAGTTCA
RIGHT PRIMER	323	18	52.51	44.44	0.00	0.00	0.00	tgatatgcagcgtgtaac

PRODUCT SIZE: 293, PAIR ANY_TH COMPL: 0.00, PAIR 3'_TH COMPL: 0.00

Guide Details
Guide: GATACGGGCCATCAAGTGGGTGG
Direction: Forwards
Restriction Enzyme (x): XCMI

Pcyt2 13 - Exon 13 Guide Design

CAGCAACAATCTGACCACTGACGACATTGTTCAGAGAATCATTGAGAACAGgtttgtaggaaaatacgagcaaaaacgctcacttga
aatatttatgaaaatatgcaacgatttttttttgtactatag[GCTGCAGTTTGAAG**CCAGGAA**[x]**TCAGAAGAA*GGAAG*CG**AAAGAAA
TGGCTGTTCTCCAGGCTTTGAAGCGTAAAGATGAATCTGTGAAAAGTGAAGCAGCTCTTGCGAAGAATTAA]CCAAACCAAGCTAAT
CGGTATTGTCGTAGAACAGAATCACCCCTGGACTCATTCAATTACATTCAGTAATGAACAGTAAGGTATTTTNAAAAAAGANAGAGG
AGGACAAGGACTTGTTTTAAATC

Primer Pair #1 (Pink)

OLIGO	start	len	tm	gc%	any_th	3'_th	hairpin	seq
LEFT PRIMER	51	21	54.97	42.86	0.00	0.00	0.00	Ggtttgtaggaaaatacgagc
RIGHT PRIMER	284	20	54.85	45.00	0.00	0.00	0.00	GGTGATTCTGTTCTACGACA

SEQUENCE SIZE: 368
INCLUDED REGION SIZE: 368

PRODUCT SIZE: 234, PAIR ANY_TH COMPL: 0.00, PAIR 3'_TH COMPL: 0.00

Primer Pair #2 (Yellow)

OLIGO	start	len	tm	gc%	any_th	3'_th	hairpin	seq
LEFT PRIMER	14	19	55.48	47.37	4.74	0.00	0.00	ACCACTGACGACATTGTTC
RIGHT PRIMER	261	18	53.41	50.00	0.00	0.00	0.00	CCGATTAGCTTGGTTTGG

PRODUCT SIZE: 248, PAIR ANY_TH COMPL: 0.00, PAIR 3'_TH COMPL: 0.00

Guide
GCTGCAGTTTGAAG**CCAGGAA**[x]**TCAGAAGAAGGAAGCG**AAAGAAATGGCTGTTCTCCAGGCTTTGAAGCGTAAAGATGAATCTGTGA
AAAGTGAAGCAGCTCTTGCGAAGAATTAA
CGCTTCCTTCTTCTGATTCCTGG
Direction: Reverse
Restriction Enzyme (x): HpyAV (recognition Site underlined as […])

cDNA primers used for zebrafish pcyt2 mRNA expression analysis

cDNA	Forward Primer	Reverse Primer
EIF1α	CTTCTCAGGCTGACTGTGC	CCGCTAGCATTACCCTCC
PCYT2 (set 1)	ACAGGCAGAGCGTTATAAAA	TTGTTAACAGGAGCATTCGA
PCYT2 (set 2)	TTTAAGGTGGATCTGGTGTG	CCATTTCTTTCGCTTCCTTC
