# Supplementary material for: Mutations in PCYT2 disrupt etherlipid biosynthesis and cause a complex hereditary spastic paraplegia
Source: Brain. 2019 Oct 22;142(11):3382–97. doi: 10.1093/brain/awz291 (PMC6821184; doi:10.1093/brain/awz291)
Supplement: awz291_Supplementary_Data [file awz291_supplementary_data.zip › awz291-Suppl_data/Supplementary_Data1.pdf]

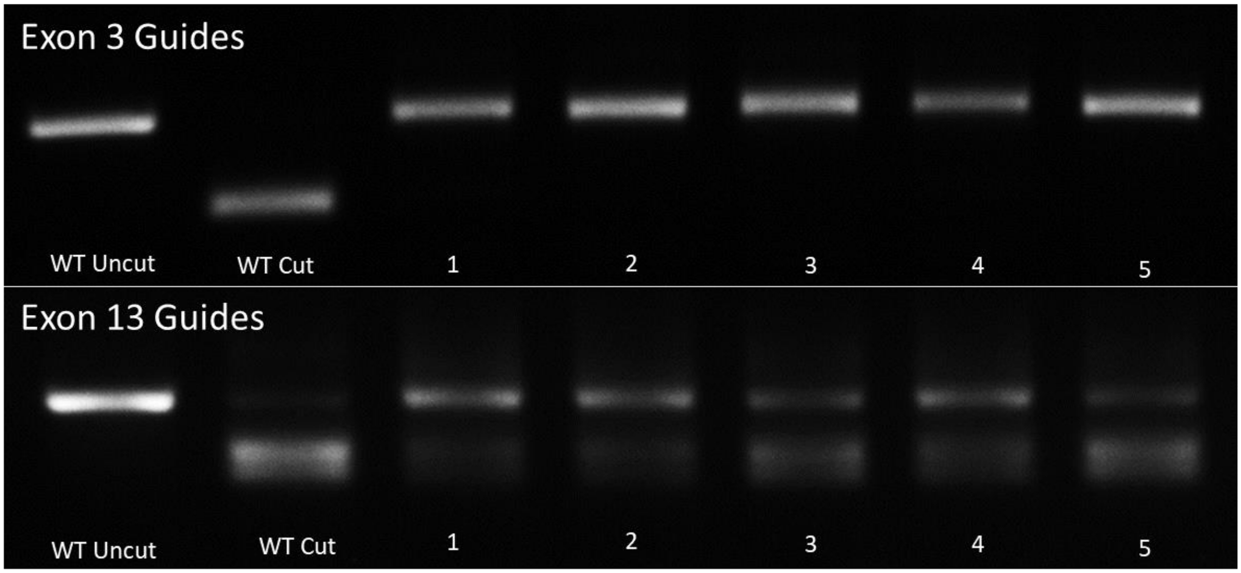

Supplementary figure 1: Zebrafish CRISPR efficiency. Restriction digest demonstrating CRISPR efficiency in day 5 zebrafish. The sgRNA were designed to target regions containing restriction enzyme sites. Efficiency of the CRISPR injections is indicated by loss of the restriction site, comparing embryos 1-5 against uncut wild type embryos and cut wild-type embryos. For guide sequences see Supplementary information 2.

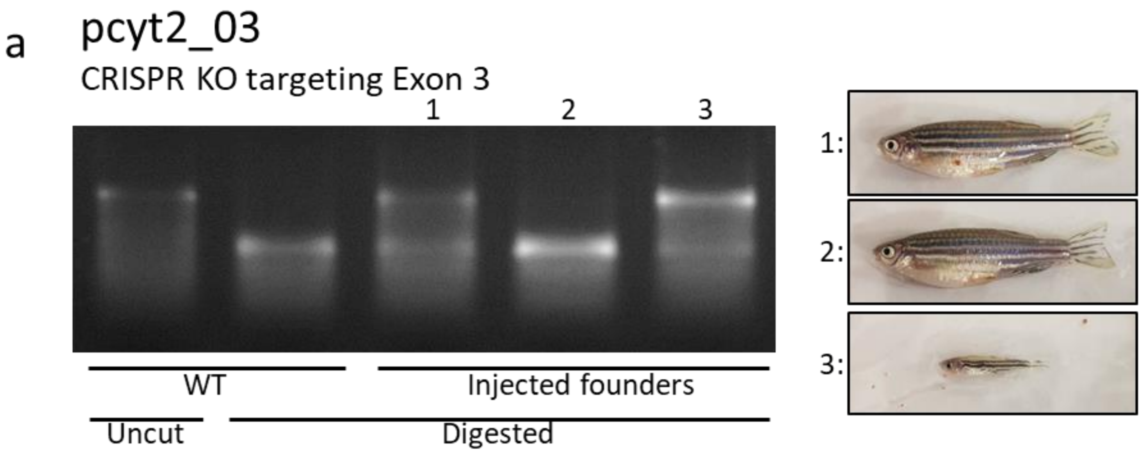

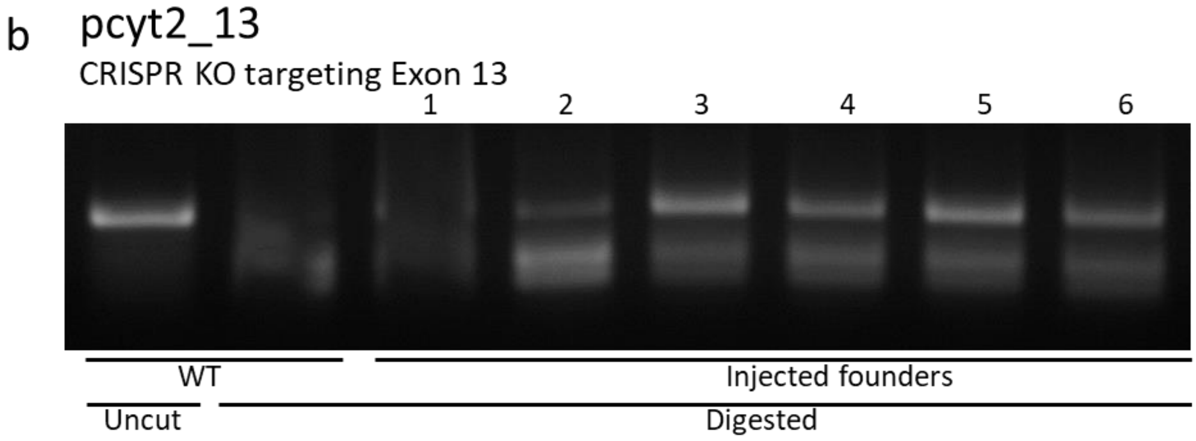

Supplementary figure 2: The efficiency of the CRISPR-CAS9 system was assessed by performing a restriction digest DNA extracted from tail-fin clippings. Good efficiency is indicated by a failure of the enzyme to produce a cut band, as this indicates that there has been complete loss of the restriction site caused by the CRISPR-CAS9 system. Poor efficiency meanwhile is demonstrated by complete cutting of the band, indicating that the restriction site is present. The mutant DNA was compared against an undigested and a digested wild-type zebrafish DNA. A) Only 3 pcyt2_03 model zebrafish survived to 6 weeks and genotyping revealed either a mosaic picture (1 & 3) or no cut at all (2). B) A sample of the pcyt2_13 models were genotyped which revealed a mosaic genotype in 5 out of the 6 samples (2-6).

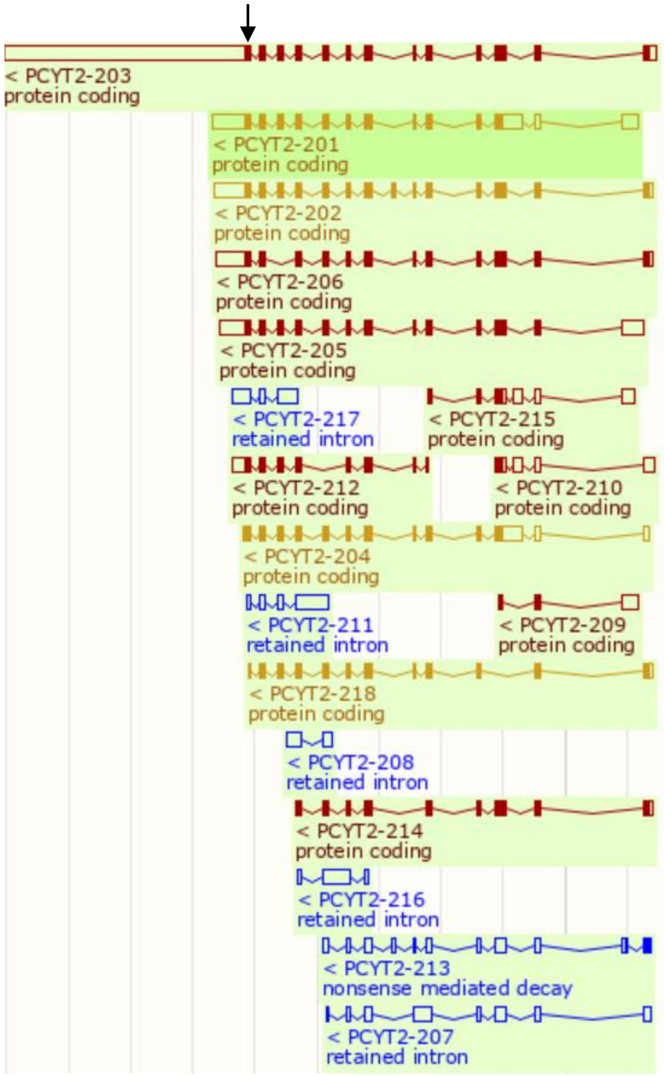

Supplementary figure 3: Transcript table detailing all known and predicted PCYT2 transcripts. PCYT2-203 represents the canonical transcript (NM_001184917) and the black arrow indicates exon 14, the region affected by the final exon nonsense variants in patients 2-5. Red transcripts indicate Ensembl protein coding transcripts. Yellow indicate protein coding transcripts predicted from Ensembl and Havana. Blue transcripts indicate non-protein coding transcripts. Figure adapted from Ensembl Genome Browser.

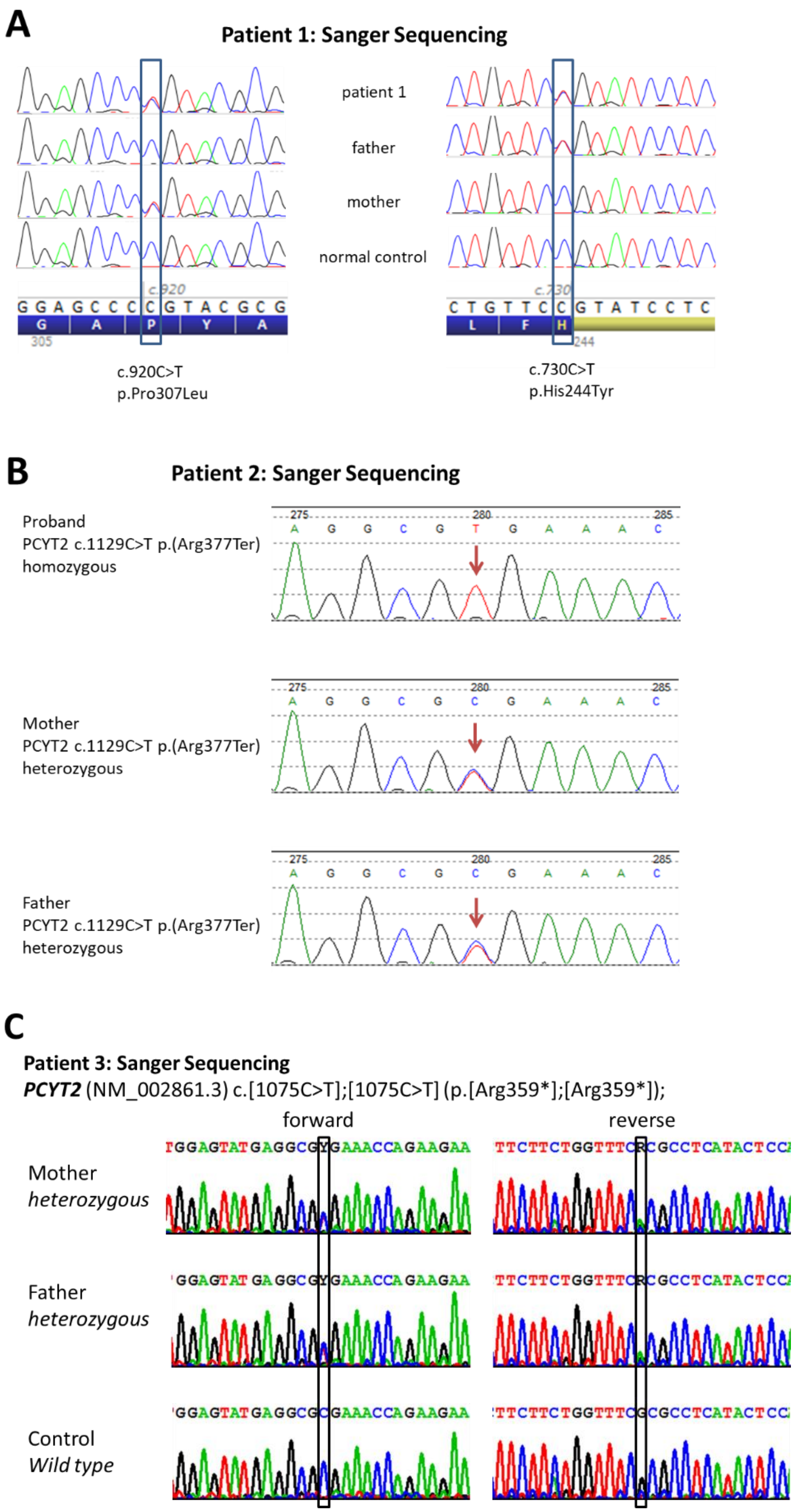

Supplementary figure 4: Sanger traces of variants for patients 1 (A) patient 1, (B) patient 2 and (C) patient 3.

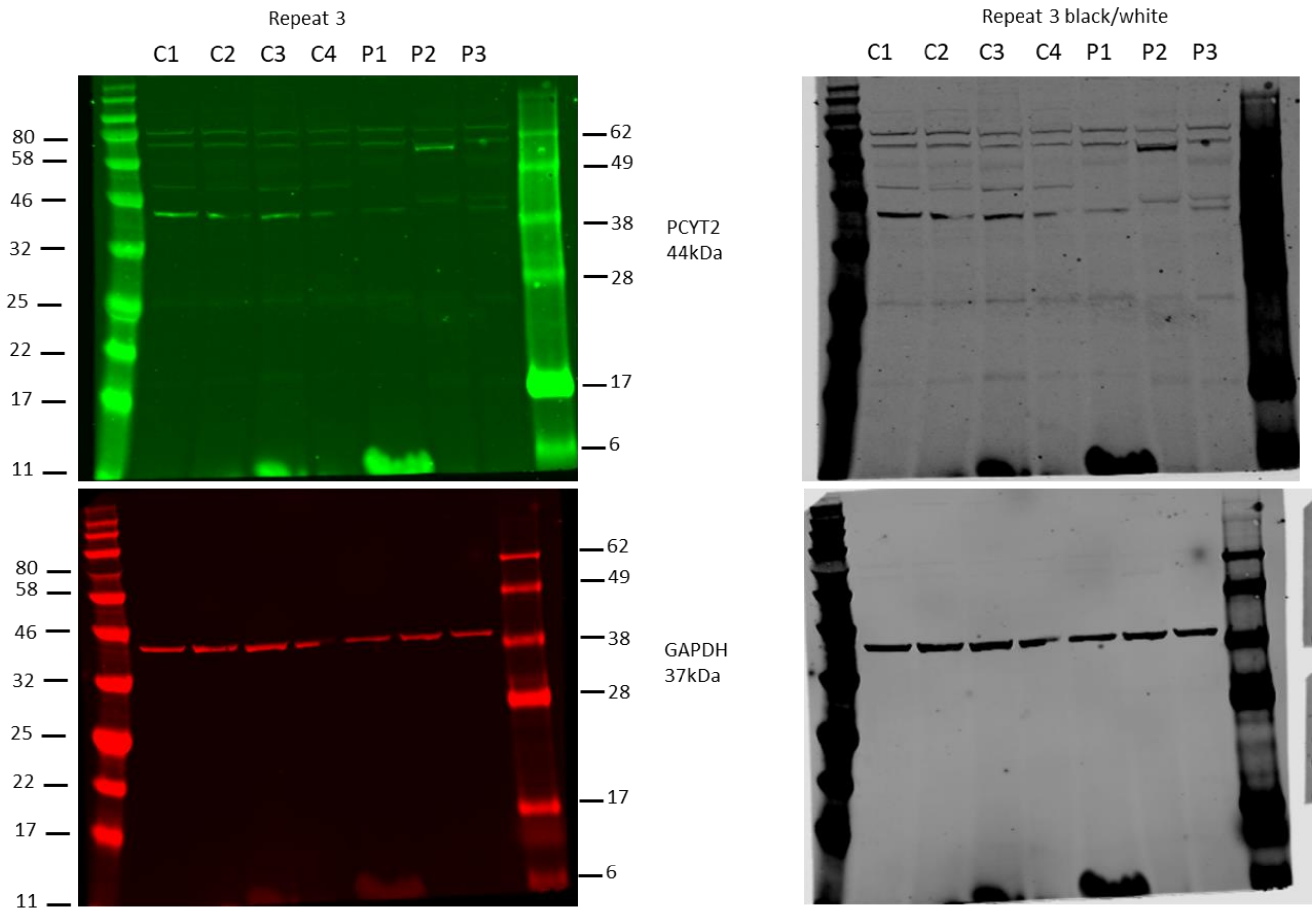

Supplementary figure 5: Complete Western Blot anti-PCYT2 and anti-GAPDH (false colour and black and white)

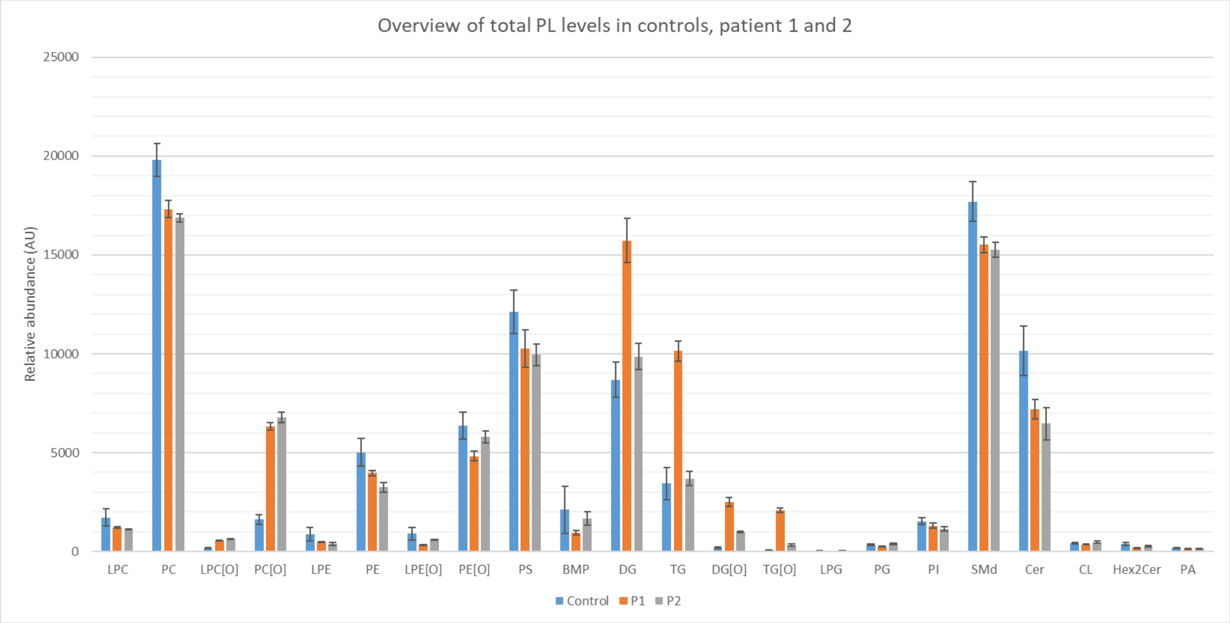

Supplementary figure 6: Overview of total phospholipid levels. Total phospholipid levels of controls, patient 1 and 2 (3 controls each measured as n=3), patient 1 (n=3) and 2 (n=4), mean +/- SD is shown) based on summed lipidomic species. Please note that comparison of amounts between different species is not possible due to the semiquantitative nature of the analysis. BMP, bis(monoacylglycero)phosphate; Cer, Ceramides; CL, cardiolipin; DG diacylglycerol; DG[O], 1-alkyl-2-acylglycerol; HexCer, hexosylceramides; LPC, lysophosphatidylchloline; LPC[O], lysoplasm(a/e)nylcholine; LPE, lysophosphatidylethanolamine; LPE[O], lysoplasm(a/e)nylethanolamine; PA, phosphatidic acid; PC, phosphatidylcholine; PC[O], plasm(a/e)nylcholine; PE, phosphatidylethanolamine; PE[O], plasm(a/e)nylethanolamine; PG, phosphatidylglycerol; PI, phosphatidylinositol; PS, phosphatidylserine; TG, triacylglycerol; TG[O], 1-alkyl-2,3-diacylglycerols.

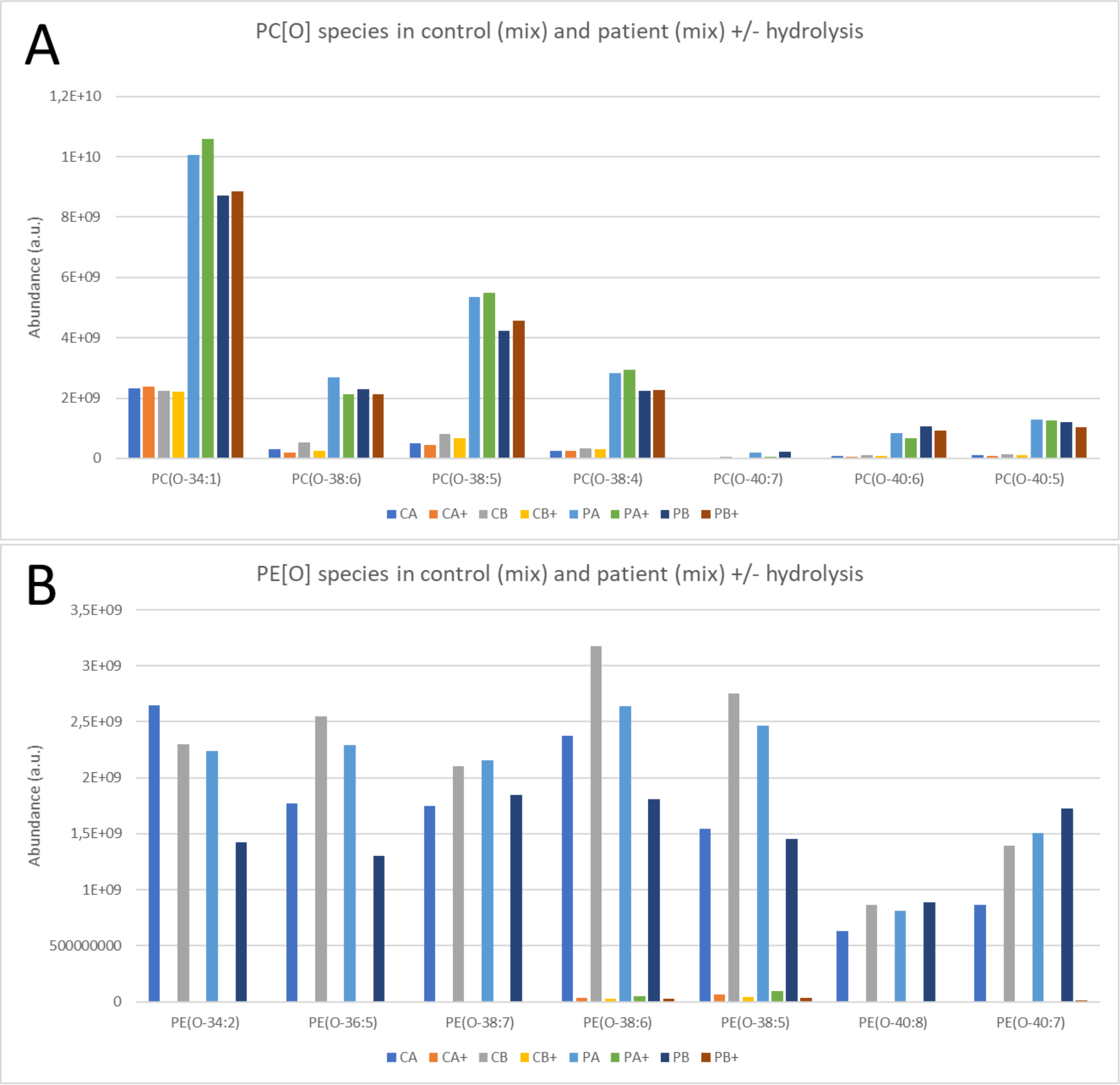

Supplementary figure 7: Plasmanyl/plasmenyl composition of selected etherphospholipid species. Plasmanyl/plasmenyl composition of (A) PC- and (B) PE-etherphospholipids: control and patient lipid extracts with and without treatment with 0.1 M HCl to hydrolyse plasmenyl species. PC-etherphospholipids are almost exclusively plasmanyl-species and PE-etherphospholipids are almost exclusively plasmenyl-species in both control and patient extracts.

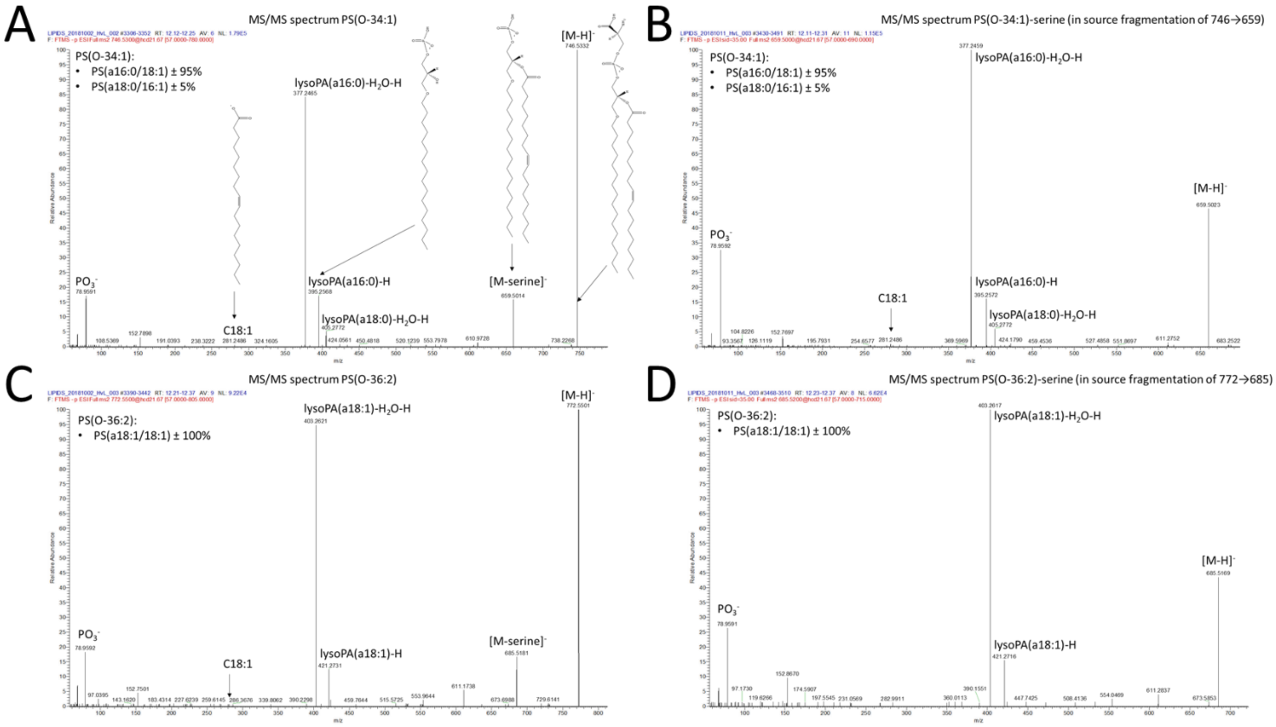

Supplementary figure 8: Fragmentation confirms the occurrence of PS[O] in *PCYT2* patients. (A) Fragmentation of m/z 746 (tentatively PS(O-34:1)) is dominated by the ions at m/z 659 (corresponding to loss of a serine head group), m/z 395 and 377 (by losses of 18:1-fatty acid substituent at sn-2 as an acid and as a ketene, respectively) and m/z 405 (loss of 16:1-fatty acid substituent at sn-2 as an acid). The spectrum also contains the ion at m/z 281, corresponding to a 18:1-carboxylate anion. The ions corresponding to the radyl substituent at sn-1 are not detected. The results show that the ion at m/z 746 is a mixture of different phosphatidylserines, this results in the structures PS(a16:0/18:1) and PS(a18:0/16:1) (where a=alkyl), with an abundancy of about 95% and 5% respectively (based on the intensities of fragments m/z 377 and 405). (B) Fragmentation of m/z 659, an in source fragmentation product of m/z 746 (tentatively PS(O-34:1) gives the same m/z 395, 377 and 405 fragments, confirming the identity of m/z 746 as PS(O-34:1). (C) Fragmentation of the ion at m/z 772 (tentatively PS(O-36:2)). This ion can be a plasmanyl-PS (a36:2) or plasmenyl-PS (p36:1). The fragmentation spectrum of m/z 772 is dominated by m/z 685 (corresponding to loss of a serine head group), m/z 403 and 421 (losses of 18:1-fatty acid substituent as an acid and as a ketene, respectively). The spectrum also contains the ion at m/z 281, corresponding to a 18:1-carboxylate anion. The results show that the ion at m/z 772 is a phosphatidylserine (PS) containing a 18:1-fatty acid substituent. (D) Fragmentation

of m/z 685, an in source fragmentation product of m/z 772 (tentatively PS(O-36:2)) gives the same m/z 403 and m/z 421 fragments. The absence of a fragment at m/z 279 (C18:2) and the ions corresponding to a radyl substituent at sn-1 supports the identification of PS(O-36:2) as the structure of PS(a36:2) = PS(a18:1/18:1) and not PS(p36:1) = PS(p18:0/18:1).

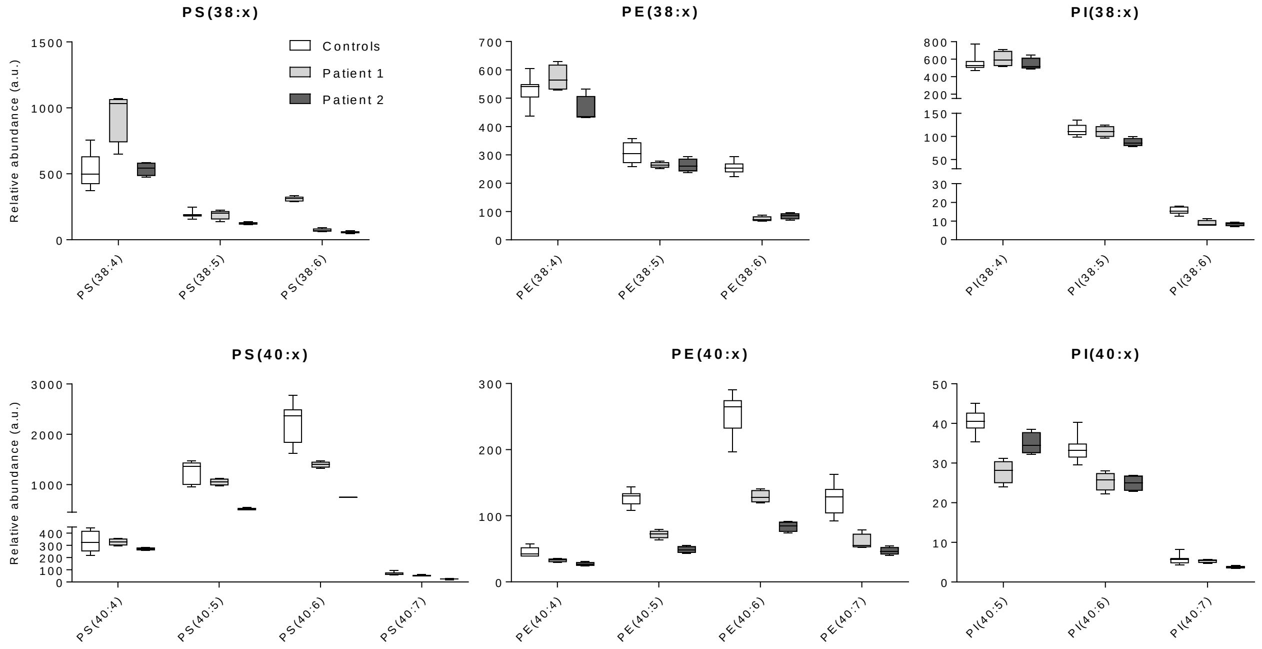

Supplementary figure 9: Levels of selected polyunsaturated phospholipid species. Box and whiskers plot of polyunsaturated species of PS, PE and PI. Species with 4 or more double bonds are shown for controls (white), patient 1 (light gray) and patient 2 (dark gray) and show a trend towards lower levels, especially for PE and PS.
